# Efficacy and safety of Chinese herbal medicines combined with biomedicine in the treatment of idiopathic membranous nephropathy: a systematic review and network meta-analysis

**DOI:** 10.3389/fphar.2024.1391675

**Published:** 2024-10-30

**Authors:** He Zhu, Yunming Xiao, Yue Ji

**Affiliations:** ^1^ Dongzhimen Hospital, Institute of Nephrology and Beijing Key Laboratory, Beijing University of Traditional Chinese Medicine, Beijing, China; ^2^ Department of Nephrology, First Medical Center of Chinese PLA General Hospital, National Key Laboratory of Kidney Diseases, Beijing Key Laboratory of Kidney Diseases Research, National Clinical Research Center for Kidney Diseases, Beijing, China

**Keywords:** idiopathic membrane nephropathy, Chinese herbal medicine, traditional Chinese medicine, network meta-analysis, randomized controlled trial

## Abstract

**Background:**

Chinese herbal medicines have been extensively used to treat idiopathic membranous nephropathy (IMN). However, their efficacy and safety remain uncertain. Therefore, this study employed a network meta-analysis to evaluate the efficacy and safety of various Chinese herbal medicines in combination with biomedicines for treating IMN.

**Methods:**

A comprehensive literature search was performed across several databases, including PubMed, EMBASE, Web of Science, Cochrane Library, China National Knowledge Infrastructure (CNKI), WanFang Data, VIP Database, and Chinese Biomedical Literature Database (CBM), to identify randomized controlled trials (RCTs) concerning the treatment of IMN using a combination of Chinese herbal medicines and biomedicine, up to 31 May 2024. Two researchers independently conducted the literature screening and data extraction. The quality of the included studies was assessed using the Cochrane quality review manual, and Stata 14.2 software was employed for network meta-analysis.

**Results:**

A total of 31 RCTs involving 2195 IMN patients and 15 different Chinese herbal medicines were analyzed. The network meta-analysis revealed that QQC + BM (84.7%) was the most effective in reducing 24-hour urinary protein. For improving serum albumin, HZC + BM (86%) was the most effective. LGT + BM (77.2%) was the best for enhancing serum creatinine levels. MXC + BM demonstrated the highest effectiveness in lowering total cholesterol (89%) and triglycerides (97%). Lastly, WZC + BM (90.8%) was the most effective in reducing the incidence of adverse reactions. BM.

**Conclusion:**

The current evidence suggests that integrating Chinese herbal medicines with biomedicine may provide significant benefits in treating IMN. Specifically, QQC + BM appears to be the most effective in reducing 24-hour urinary protein, HZC + BM seems to excel in improving serum albumin levels, MXC + BM is noted for its effectiveness in lowering triglycerides and total cholesterol, LGT + BM is optimal for reducing serum creatinine, and WZC + BM shows the lowest rate of adverse reactions. Nevertheless, due to limitations in the quantity and quality of the included studies, further validation of these conclusions is necessary.

**Systematic Review Registration:**

[https://www.crd.york.ac.uk/prospero/display_record.php?ID=CRD42024561028], identifier [CRD42024561028].

## 1 Introduction

Idiopathic membranous nephropathy (IMN) is a glomerular disease marked by the thickening of the basement membrane, manifesting clinically with significant proteinuria, hypoproteinemia, edema, and hyperlipidemia (Xue et al., 2023). It is a prevalent cause of nephrotic syndrome (Liu et al., 2020). In recent years, the incidence of IMN has significantly risen in our country, ranking it as the second most common primary glomerular disease (Hou et al., 2018). Studies have indicated that some IMN patients undergo spontaneous remission, while a significant number progress to end-stage renal disease (ESRD) (Dahan et al., 2017). International guidelines recommend immunotherapy as the primary treatment, although its side effects present challenges in clinical practice (Wu et al., 2021). A considerable proportion of patients encounter sensitivity, adverse reactions, and intolerance to combined hormone and immunosuppressive therapy (Xiang et al., 2022). The traditional clinical treatment involves the use of immunosuppressants combined with glucocorticoids, such as cyclophosphamide with methylprednisolone, or cyclosporin A with low-dose prednisone (Xue et al., 2023; Chen et al., 2020). However, prolonged use of these medications can lead to metabolic disorders, infections, bone marrow suppression, and other adverse effects (Shan et al., 2024). Newer biological agents like monoclonal antibodies can effectively inhibit autoimmune responses and avoid side effects such as kidney damage (van den Brand et al., 2017), but their high cost makes them inaccessible to many patients (Shan et al., 2024). Thus, reducing treatment-related adverse reactions and enhancing the clinical therapeutic effect of IMN remains a significant clinical challenge.

Based on clinical symptoms of IMN, such as proteinuria and edema, it is commonly classified under the categories of “turbidity” and “edema” in traditional Chinese medicine (Lu et al., 2021). Chinese medicine offers significant benefits in the treatment of IMN, enhancing efficacy and reducing toxicity (Yang et al., 2023). Chinese herbal medicines are extensively used in clinical practice due to their multi-target approach, ease of use, and minimal side effects (Lu et al., 2019). Traditional Chinese medicine (TCM) has become widely accepted in the clinical management of IMN due to its proven effectiveness (Yang et al., 2023). Current clinical data indicates that combining traditional Chinese medicine with biomedicine produces better results than using biomedicine alone (Yang et al., 2023). Nevertheless, the absence of direct comparative evidence has sparked debate over the differing efficacies of various traditional Chinese medicine regimens. Network meta-analysis, an innovative method for integrating clinical data, provides a way to assess treatment efficacy by combining direct and indirect comparisons (Cortese et al., 2018). This study employed network meta-analysis to develop a comprehensive model for comparing various treatment options that incorporated both traditional Chinese medicine and biomedicine, aiming to offer valuable insights for clinical decision-making in healthcare environments.

## 2 Methods

### 2.1 Inclusion and exclusion criteria

For this research, only studies that met the following criteria were included:

(1) Study design: restricted to randomized controlled trials (RCTs) as the research type, with languages confined to Chinese and English.
(2) Disease: participants in this study were patients diagnosed with IMN, with no limitations on gender, age, or disease duration, etc.
(3) Intervention: The intervention group in this study received a combination of conventional biomedicine and traditional Chinese medicine, including proprietary Chinese medicinal products and extracts. To maintain consistency, the study excluded any homemade or in-hospital prepared traditional Chinese medicines. The biomedicine treatments included commonly used clinical medications (such as glucocorticoids, immunosuppressants, and renin-angiotensin system inhibitors), without specific restrictions on the treatment regimen. The control group was administered either standard biomedicine or a placebo, with the biomedicine protocol being identical to that of the intervention group.
(4) Outcomes: Primary outcome indicators: 24-hour urine protein; Secondary outcome indicators: ① Serum albumin; ② Blood creatinine; ③ Total cholesterol; triglycerides; Adverse reactions.

Studies that met the following criteria were excluded:

(1) The research was republished;
(2) The research data was evidently inaccurate or incomplete, and efforts to obtain the necessary data from the author failed;
(3) The experimental group in the studies included a variety of TCM-based treatments.

### 2.2 Search strategy

A comprehensive search was carried out in multiple databases, including PubMed, EMBASE, Web of Science, Cochrane Library, China Knowledge Network (CNKI), WanFang Database, VIP Database, and China Biomedical Literature Database. The objective was to identify RCTs that investigated the treatment of IMN with a combination of traditional Chinese medicine and biomedicine. The search covered the period from the inception of each database until 31 May 2024. Search methods employed both keywords and free-text terms tailored to the specific search features of each database. In the Chinese databases, keywords such as “te fa xing mo xing shen bing” (idiopathic membrane nephropathy) and “zhong yao” (traditional Chinese medicine) were employed, while the free text search included terms like “mo xing shen bing” (membrane nephropathy), “zhong yi yao” (traditional Chinese medicine), “zhong yao fu fang” (traditional Chinese medicine compound), “zhong cheng yao” (proprietary Chinese medicine), “zhu she ji” (injection), “jiao nang” (capsule), and “pian ji” (tablet). In the English databases, topics such as “glomerulonephritis, Membrane” and “Chinese medicine” were used, alongside free terms like “Membrane glomerulonephritis,” “Heymann nephritis,” “Idiopathic membrane glomerulonephritides,” “Membrane nephropathy,” “traditional Chinese medicine,” “Chinese polyherbal preparation (CCPP),” “Oral liquid,” and “Chinese polyherbal preparation (CCPP),” among others. The Supplementary Material provide more information about the search strategy using Pubmed as an example (Supplementary Table S1). The study protocol was registered on PROSPERO (CRD 42024561028).

### 2.3 Literature screening and data extraction

The study utilized the EndNote X7 software for document management to independently filter documents, which started by removing duplicates, then excluded clearly unqualified research based on titles and abstracts, and finally refined the remaining research by reviewing the full texts. Throughout the screening process, two researchers, Ji Yue and Zhu He, independently examined the literature. They then cross-checked the screening results, discussing and assessing any research items that presented discrepancies or challenges in determining their inclusion status. When needed, a third researcher was consulted. Additionally, pertinent information was extracted using Excel 2013, including: ① clinical research details (such as the title, lead author, year of publication, sample size, and average age), ② intervention details (including types, dosages, and duration of biomedicine in the control group, and dosages, frequency, and duration of TCM in the intervention group), ③ various elements for assessing bias risks in RCTs and outcome indicators (where continuous variable data involved calculating the difference between pre- and post-treatment changes, specifically the mean and standard deviation variances).

### 2.4 Bias risk assessment included in the study

The quality assessment was conducted by two researchers using the Cochrane System Evaluation Manual Version 5.1.0 RCT bias risk assessment tool to assess the relevant literature concurrently. If there was a disagreement between the two researchers, a third researcher was consulted for his/her opinion. The evaluation criteria included aspects such as random sequence generation, allocation concealment, blinding procedures for participants and assessors, blinding of outcome evaluators, incomplete outcome data, selective reporting, and other potential biases (Higgins and Green, 2008).

### 2.5 Statistical analysis

The study utilized Stata14.2 software (StataCorp, College Station, TX) to create an evidence network diagram illustrating the comparative relationships among interventions for each outcome indicator. Binary variables’ effect measures were denoted by relative risk (RR), while continuous variables were represented by Weighted Mean Difference (BMD) as effect indicators, with each effect estimate provided alongside its 95% confidence interval. The I^2^ value was used to assess the heterogeneity among the included studies. If *P* > 0.1 and I^2^ ≤ 50%, it was interpreted that there was no significant statistical heterogeneity, and a fixed-effect model was applied for the meta-analysis. Conversely, if *P* ≤ 0.1 and I^2^ > 50%, the studies were considered heterogeneous, prompting the use of a random-effects model. Subgroup analysis was performed based on treatment duration (4 months, 6 months, and 12 months). Sensitivity analysis was performed using one-by-one elimination method. A comparison-adjusted funnel plot was employed to detec t possible small-study effects and evaluate publication bias. The efficacy of interventions for each outcome measure was ranked using the Surface Under the Cumulative Ranking (SUCRA) values derived from the cumulative ranking curve.

## 3 Results

### 3.1 Literature search results

**FIGURE 1:**
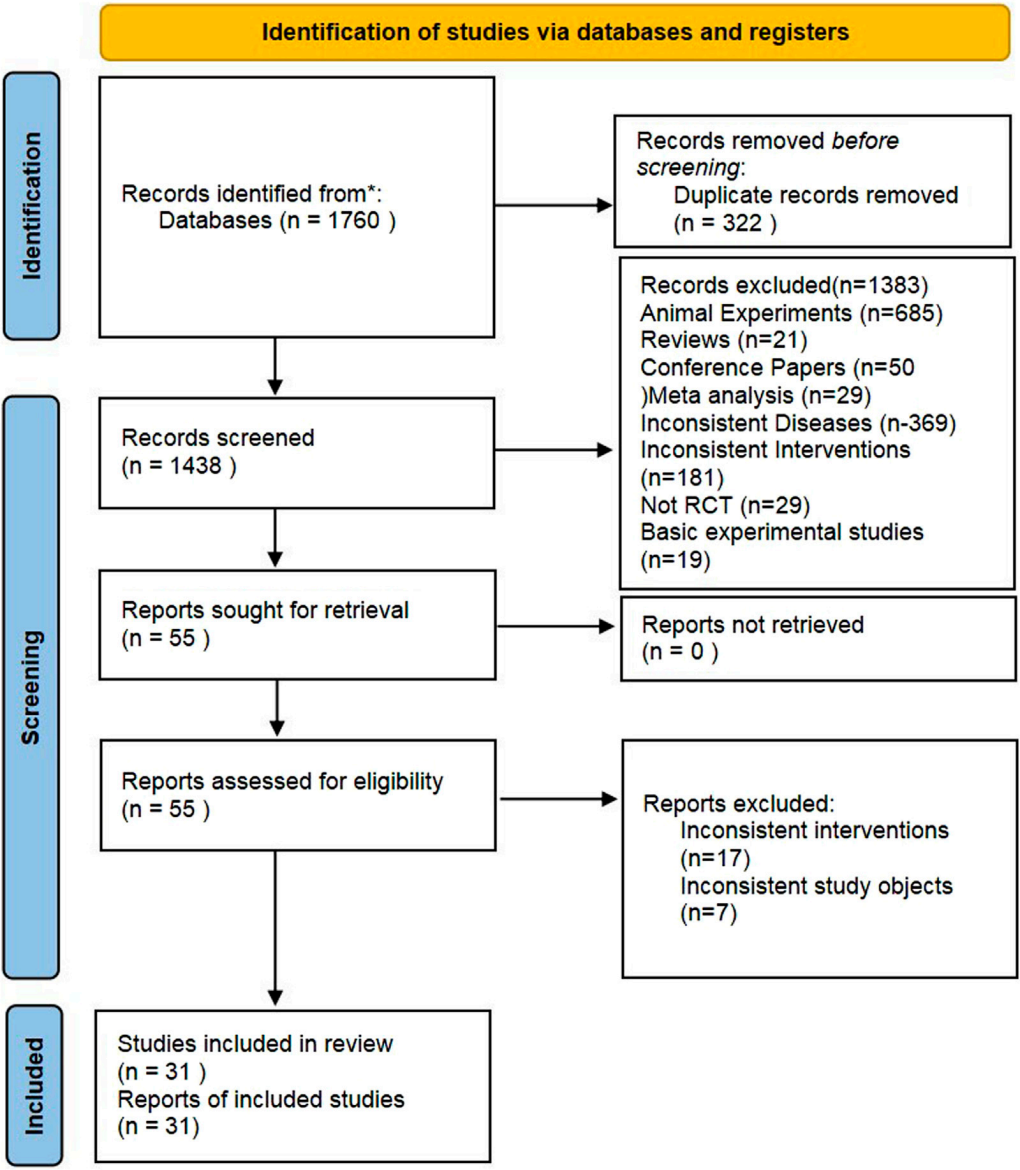
Literature screening process.

In this study, a total of 1,760 pertinent documents were retrieved. After an initial and a subsequent re-screening, 31 RCTs (Sun et al., 2014; Chen and Mei, 2015; Chen et al., 2022; Cui and Li, 2020; Fei et al., 2021; Feng et al., 2015; Gao, 2013; Gao et al., 2014; Hao and Yu, 2017; Li et al., 2021; Liu et al., 2016; Lu and Ren, 2019; Lv et al., 2020; Ma et al., 2014; Wang, 2020; Zhu et al., 2020; Cheng et al., 2017; Cui et al., 2013; Gan, 2016; Guo et al., 2013; Ling et al., 2015; Liu et al., 2012; Liu, 2015; Ping et al., 2021; Xie and Dang, 2016; Feng et al., 2013; Han et al., 2015; Lv et al., 2017; Pan et al., 2016; Ren et al., 2013; Xu and Xu, 2021) were identified. Ultimately, 2,195 patients were included in the study, which covered various treatments like Wuzhi capsules (WZC), Leigongteng polysaccharide tablets (LGT), Shengmai Injection (SMI), Shenfukang II capsules (SFC), Huangzhi Yishen capsules (HZC), Maixuekang capsules (MXC), Kunxian capsules (KXC), Bailing capsules (BLC), Dihuang Ye Total Glycoside capsules (DHT), Qing Re Mo Shen granules (QRG), Shen Yan Kang Fu tablets (SYT), Qiqi Yi Shen capsules (QQC), Huo Ba Hua Gen tablets (HBT), Renkang Injection (SKI), Huangkui capsules (HKC), and biomedicine (BM). The literature screening process is illustrated in Figure 1.

### 3.2 The basic characteristics of the studies

Among the 31 included studies, 16 articles (Sun et al., 2014; Chen and Mei, 2015; Chen et al., 2022; Cui and Li, 2020; Fei et al., 2021; Feng et al., 2015; Gao, 2013; Gao et al., 2014; Hao and Yu, 2017; Li et al., 2021; Liu et al., 2016; Lu and Ren, 2019; Lv et al., 2020; Ma et al., 2014; Wang, 2020; Zhu et al., 2020) involved the use of a combination of TCM and immunosuppressants, 9 articles (Xiang et al., 2022; Cheng et al., 2017; Cui et al., 2013; Gan, 2016; Guo et al., 2013; Ling et al., 2015; Liu et al., 2012; Liu, 2015; Ping et al., 2021; Xie and Dang, 2016) examined the combination with ARBs (Angiotensin II Receptor Blockers), and 6 articles (Feng et al., 2013; Han et al., 2015; Lv et al., 2017; Pan et al., 2016; Ren et al., 2013; Xu and Xu, 2021) explored the combination with ACEIs (Angiotensin-Converting Enzyme Inhibitors). Twenty-nine studies (Sun et al., 2014; Chen and Mei, 2015; Chen et al., 2022; Cui and Li, 2020; Fei et al., 2021; Feng et al., 2015; Gao, 2013; Gao et al., 2014; Hao and Yu, 2017; Li et al., 2021; Liu et al., 2016; Lu and Ren, 2019; Lv et al., 2020; Ma et al., 2014; Wang, 2020; Zhu et al., 2020; Cheng et al., 2017; Cui et al., 2013; Gan, 2016; Guo et al., 2013; Ling et al., 2015; Liu et al., 2012; Ping et al., 2021; Xie and Dang, 2016; Feng et al., 2013; Han et al., 2015; Lv et al., 2017; Pan et al., 2016; Xu and Xu, 2021) reported 24-hour urinary protein levels, 26 studies (Chen and Mei, 2015; Chen et al., 2022; Cui and Li, 2020; Feng et al., 2015; Gao, 2013; Gao et al., 2014; Hao and Yu, 2017; Li et al., 2021; Liu et al., 2016; Lu and Ren, 2019; Lv et al., 2020; Ma et al., 2014; Wang, 2020; Zhu et al., 2020; Cheng et al., 2017; Cui et al., 2013; Gan, 2016; Guo et al., 2013; Ling et al., 2015; Liu et al., 2012; Ping et al., 2021; Xie and Dang, 2016; Feng et al., 2013; Han et al., 2015; Pan et al., 2016; Xu and Xu, 2021) reported serum albumin levels, and 20 studies (Sun et al., 2014; Chen and Mei, 2015; Chen et al., 2022; Cui and Li, 2020; Gao, 2013; Hao and Yu, 2017; Li et al., 2021; Lu and Ren, 2019; Wang, 2020; Zhu et al., 2020; Cui et al., 2013; Gan, 2016; Guo et al., 2013; Liu et al., 2012; Ping et al., 2021; Xie and Dang, 2016; Feng et al., 2013; Han et al., 2015; Pan et al., 2016; Xu and Xu, 2021) reported serum creatinine levels. Additionally, 11 studies (Feng et al., 2015; Gao et al., 2014; Hao and Yu, 2017; Liu et al., 2016; Ma et al., 2014; Wang, 2020; Gan, 2016; Ling et al., 2015; Xie and Dang, 2016; Han et al., 2015; Xu and Xu, 2021) reported total cholesterol levels, 10 studies (Feng et al., 2015; Gao et al., 2014; Liu et al., 2016; Ma et al., 2014; Wang, 2020; Gan, 2016; Ling et al., 2015; Xie and Dang, 2016; Han et al., 2015; Xu and Xu, 2021) reported triglyceride levels, and 16 studies (Chen and Mei, 2015; Chen et al., 2022; Cui and Li, 2020; Feng et al., 2015; Gao, 2013; Hao and Yu, 2017; Li et al., 2021; Lv et al., 2020; Wang, 2020; Zhu et al., 2020; Guo et al., 2013; Ling et al., 2015; Liu, 2015; Xie and Dang, 2016; Feng et al., 2013; Ren et al., 2013) reported adverse reactions. Table 1 displays the fundamental characteristics of the included studies, while Table 2 outlines the intervention measures.

**TABLE 1:** The basic characteristics of included studies.

Study	Number of cases (T/C)	Gender (male/female)		Age (years old)		Course of illness (month)	
		T	C	T	C	T	C
Sun et al. (2014)	30/30	17/13	9/11	40.15 ± 10.05	39.37 ± 11.73	12.50 ± 5.00	11.00 ± 4.50
Chen and Mei (2015)	21/27	14/7	15/12	56.10 ± 10.00	52.30 ± 12.90	unclear	unclear
Chen (2022)	49/48	32/17	30/18	57.01 ± 5.22	56.02 ± 5.17	24.00 ± 6.07	22.50 ± 6.02
Cui and Li (2020)	30/30	14/16	15/15	48.10 ± 8.50	49.20 ± 8.40	10.20 ± 1.60	11.40 ± 1.20
Fei et al. (2021)	28/25	16/12	13/12	62.61 ± 9.44	62.80 ± 8.82	8.35 ± 1.31	8.30 ± 1.27
Feng et al. (2015)	120/120	74/46	76/44	45.6 ± 10.4	46.20 ± 9.50	2.50 ± 1.50	2.60 ± 1.60
Gao (2013)	15/15	unclear	unclear	unclear	unclear	unclear	unclear
Gao et al. (2014)	33/27	20/13	16/11	41.05 ± 18.04	47.40 ± 7.95	unclear	unclear
Hao and Yu (2017)	32/32	unclear	unclear	unclear	unclear	unclear	unclear
Li et al. (2021)	54/53	35/19	33/20	42.98 ± 2.49	43.26 ± 2.50	16.61 ± 1.89	16.57 ± 1.80
Liu et al. (2016)	40/34	18/22	18/16	41.20 ± 9.20	43.10 ± 7.70	unclear	unclear
Lu and Ren (2019)	107/105	75/32	72/33	62.80 ± 9.20	63.20 ± 9.90	13.20 ± 1.70	13.70 ± 2.10
Lv (2020)	20/20	16/4	14/6	44.45 ± 19.04	40.15 ± 17.11	4.00 ± 1.86	3.45 ± 1.79
Ma et al. (2014)	30/30	16/14	16/14	15∼65	17∼60	unclear	unclear
Wang (2020)	38/38	unclear	unclear	unclear	unclear	unclear	unclear
Zhu et al. (2020)	25/25	13/12	16/9	48.28 ± 3.25	47.58 ± 3.17	8.23 ± 2.88	8.42 ± 2.91
Cheng et al. (2017)	24/26	13/11	14/12	49.28 ± 14.20	48.51 ± 15.21	unclear	unclear
Cui et al. (2013)	38/38	22/16	24/14	52.30 ± 3.70	51.90 ± 3.40	2.80 ± 0.30	2.40 ± 0.30
Gan (2016)	31/32	18/13	17/15	53.74 ± 11.65	44.53 ± 15.60	28.53 ± 53.41	25.81 ± 39.75
Guo (2013)	24/24	unclear	unclear	unclear	unclear	unclear	unclear
Ling et al. (2015)	42/43	20/22	21/22	43.12 ± 2.68	42.87 ± 3.49	8.35 ± 2.47	8.13 ± 2.35
Liu et al. (2012)	30/30	20/10	15/15	55.30 ± 12.40	54.00 ± 13.20	11.40 ± 8.20	12.30 ± 9.50
Liu (2015)	28/28	16/12	14/14	33∼73	32∼72	0.1∼36	0.1∼36
Ping et al. (2021)	40/40	21/19	22/18	41.30 ± 10.20	40.30 ± 11.10	11.20 ± 4.70	10.30 ± 5.10
Xie Dang (2016)	30/30	18/12	18/12	33∼73	32∼72	0.1∼36	0.1∼36
Feng et al. (2013)	21/20	10/11	11/9	18∼54	20∼50	0.5∼6	0.5∼6
Han et al. (2015)	25/25	unclear	unclear	44.60 ± 6.30	46.40 ± 6.10	5.00 ± 3.20	5.40 ± 2.90
Lv (2017)	35/32	21/14	19/13	38.50 ± 1.90	39.30 ± 2.60	unclear	unclear
Pan et al. (2016)	25/25	12/13	14/11	44.6 ± 6.3	46.40 ± 6.10	5.00 ± 3.20	5.40 ± 2.90
Ren et al. (2013)	30/30	unclear	unclear	40.10 ± 10.0	39.30+11.73	12.50 ± 5.00	11.00+4.50
Xu and Xu (2021)	40/40	24/16	23/17	48.12 ± 10.51	47.45 ± 10.33	5.84 ± 2.36	5.94 ± 2.26

**TABLE 2:** Characteristics of interventions in the study.

Author	Research type	Interventions		Treatment	Outcome indicator
		Chinese medicine program	Biomedicine program		
Sun et al. (2014)	Two arms	Wuzhi capsules: 1 capsule, Tid, can be gradually increased to a maximum of 3 capsules, Tid	Tacrolimus capsules: 0.05 mg/kg/d, Bid, gradually increase to a blood concentration of 4∼8 ng/mL; prednisone 30 mg/day, reduce by 5 mg every 4 weeks after 8 weeks, and maintain after reducing to 10 mg/d	6 months	①②
Chen and Mei (2015)	Two arms	Wuzhi capsules: 2 capsules/time, 2 times/d	Xinsaisepine: 2.5∼3.0 mg (kg·d), Bid; prednisone tablets: 0.5 mg (kg·d), QD	6 months	①②③
Chen et al. (2022)	Two arms	Lei Gongteng Polysaccharide Tablets: 20 mg/time, 3 times/d	Tacrolimus capsules: 0.05 mg/(kg·d) Bid; prednisone tablets: 1 mg/(kg·d), reduce by 5 mg/d every 2 weeks, reduce to 10 mg/d and reduce to 2.5 mg/d in less than 2 weeks	6 months	①②③⑥
Cui and Li (2020)	Two arms	Lei Gongteng Polysaccharide Tablets: 60 mg/d, Tid	Prednisone tablets: 1 mg/(kg·d), start to reduce the amount by 5 mg/d every 2 weeks after 2 weeks, and after reducing to 10 mg/d, reduce to 2.5 mg/d every 2 weeks until the drug is discontinued; Tacrolimus capsules: 0.05 mg/(kg·d), Bid	6 months	①②③⑥
Fei et al. (2021)	Two arms	Shengmai injection: Add 250 mL of saline to 40 mL, 1 time/d, 1 course of treatment for 14 d and 16 d for 1 course of treatment, a total of 6 courses of treatment.	Prednisone tablets: 1 mg/kg, Qd, after 8 weeks, reduce the amount every 2 weeks to 10 mg/d to maintain; cyclophosphamide for injection: 600 mg/month for the first month, 800 mg/time for the next month, 1 time/month	6 months	①⑥
Feng et al. (2015)	Two arms	Lei Gongteng Polysaccharide Tablets: 20 mg/time, Tid	Prednisone tablets: 0.5 mg/(kg·d), the maximum dose is 30 mg/d, after 8 weeks, the amount is reduced by 5 mg every 2 weeks and every other day, and the amount is gradually reduced to 10 mg/d after 6 months	6 months	①③④⑤⑥
Gao (2013)	Two arms	Lei Gongteng polysaccharide tablets 1 mg/(kg·d), the maximum dose is 60 mg/d, maintained for 6 months, according to the patient's condition can be reduced to 30 mg/d	Tacrolimus capsules: 0.05 m/(kg·d), Bid, after 2 weeks, increase the dosage according to the blood concentration, no more than 0.1 mg/(kg·d); prednisolone: 0.5 mg/(kg·d), the maximum dose is 30 mg/d, after 8 weeks, reduce by 5 mg every 2 weeks every other day, and maintain it at 10 mg/d after 6 months	6 months	①②③⑥
Gao et al. (2014)	Two arms	Shenfukang II Capsules: 1 Dose/d, Bid	Prednisone; 1 mg/(kg·d), Qd, 5 mg per month after 8 to 12 weeks of continuous use, reduced to 0.5 mg/(kg·d), 5 mg per month after 2 to 3 months of continuous use, reduced to 0.2 mg/(kg·d), taken for 6 months; cyclophosphamide: 15∼20 mg/kg each time, adult 1.0 g/month, add 500 ml of saline intravenously	6 months	①③④⑤
Hao and Yu (2017)	Two arms	Huangzhi Yishen Capsules: 5 Capsules/time, Tid	Prednisone tablets: 40 mg/time/d, Qd, continuous use for 12 weeks to gradually reduce the amount; or injection of cyclophosphamide 200 mg + 0.9% sodium chloride 20 mL intravenous bolus, once every other day, the total dose is less than 8 g	6 months	①②③④⑥
Li et al. (2021)	Two arms	Lei Gongteng Polysaccharide Tablets: 20 mg/time, Tid	Tacrolimus capsules: 0.05 mg/time, Bid	4 months	①②③⑥
Liu et al. (2016)	Two arms	Maixuekang Capsules: 1 g, Tid	Prednisone: 1 mg/(kg·d); cyclophosphamide: 0.6 g/time, once every 2 weeks	4 weeks	①③④⑤
Lu and Ren (2019)	Two arms	Kunxian Capsules: 0.6 g/time, Tid	Prednisone tablets: 5 mg/time, after 2 months of oral administration, reduce the dose by 5 mg/d every 2 weeks and every other day until the amount is reduced to 10 mg/d	6 months	①②③
Lv et al. (2020)	Two arms	Bailing Capsules: 2 g/time, Tid	Prednisone tablets: 1 mg/(kg·d), reduce the original dose by 10% every 2 weeks after 8 weeks; cyclophosphamide: 0.8∼1 g/time, 1 time/month, discontinue after accumulating 7∼8 g	3 months	①③⑥
Ma et al. (2014)	Two arms	Leigongteng Polysaccharide Tablets: 20 mg, Tid	Prednisone tablets: 1 mg/(kg·d), Qd, reduce the amount after 8 weeks, reduce the total amount by 10 every 2 weeks%	12 weeks	①③④⑤
Wang (2020)	Two arms	Lei Gongteng Polysaccharide Tablets: 40 mg, Tid, reduced to 60 mg/d after 3 months	Tacrolimus capsules: 0.05 mg/(kg·d), Bid, maintain blood concentration at (4∼10) ng/mL; Prednisone tablets: 0.5 mg/(kg·d), Qd, gradually reduce the amount to discontinuation	12 months	①②③④⑤⑥
Zhu et al. (2020)	Two arms	Lei Gongteng polysaccharide tablets: 1 mg/(kg·d), tid, start to reduce the dose by 10% every two weeks after 2 months, and continue to take the medicine at a dose of 10∼20 mg/d for 6 months	Prednisone tablets: 1 mg/(kg·d) Qd, reduce the dose by 10% every two weeks from 2 to 3 months. When the dose is reduced to 0.5 mg/kg·d, continue to take the medicine at a dose of 5 to 10 mg/d for 6 months	12 months	①②③⑥
Cheng et al. (2017)	Two arms	Dihuang Ye Total Glycoside Capsules: 2 capsules/time, Bid	Candesartan ester tablets: 4 mg, Qd	8 weeks	①③
Cui et al. (2013)	Two arms	Bailing Capsules: 2 g/time, Tid	Erbesartan tablets: 150 mg, Qd	3 months	①②③
Gan (2016)	Two arms	Qing Re Mo Shen granules: 12 g/pack, 1 pack/time, Tid	Losartan potassium tablets: 50 mg, Qd; heat-clearing membrane kidney particle simulator: 12 g, Tid	6 months	①②③④⑤
Guo (2013)	Two arms	Shen Yan Kang Fu tablets: 0.48 g/tablet, 5 tablets/time, Tid	Losartan potassium tablets: 50 mg, Qd	6 months	①②③⑥
Ling et al. (2015)	Two arms	Lei Gongteng Polysaccharide tablets: 40 mg, Tid, reduce the dose by 10 per week after 3 months%	Desartan capsules: 80 mg, Qd, if there are no obvious adverse reactions, increase the dose to 160 mg, Qd	December	①③④⑤⑥
Liu et al. (2012)	Two arms	Shen Yan Kang Fu tablets: 0.48 g/tablet, 5 tablets/time, Tid	Valsartan capsules: 160 mg, Qd	6 months	①②③⑥
Liu (2015)	Two arms	Huangkui Capsules: 0.5 g/capsule, 5 capsules/time, Tid	Erbesartan tablets: 150 mg, Bid	12 weeks	⑥
Ping et al. (2021)	Two arms	Qiqi Yi Shen Capsules: 4 Capsules, Tid	Losartan potassium tablets: 50 mg∼100 mg/day	16 weeks	①②③
Xie and Dang (2016)	Two arms	Huo Ba Hua Gen tablets: 0.18 g/tablet, 3∼5 tablets/time, Tid	Erbesartan tablets: 150 mg, Bid	12 weeks	①②③④⑤⑥
Feng et al. (2013)	Two arms	Renkang Injection 100 mL +5% glucose Injection 250 mL, Qd	Benapril Hydrochloride tablets: 10 mg, Bid	4 weeks	①②③⑥
Han et al. (2015)	Two arms	Huangkui Capsules: 0.5 g/capsule, 5 capsules/time, Tid	Benapril Hydrochloride tablets: 10 mg, Qd	8 weeks	①②③④⑤
Lv (2017)	Two arms	Huangkui Capsules: 0.5 g/capsule, 5 capsules/time, Tid	Benapril Hydrochloride tablets: 10 mg, Bid	8 weeks	①
Pan et al. (2016)	Two arms	Huangkui Capsules: 0.5 g/capsule, 5 capsules/time, Tid	Benapril Hydrochloride tablets: 10 mg, Qd	8 weeks	①②③
Ren et al. (2013)	Two arms	Wuzhi capsules: 1 capsule, Tid	Tacrolimus capsules: The initial dose is 0.05 mg/(kg·d), Bid, and the dose is gradually increased to maintain a blood concentration valley of 4∼8 µg/L	unclear	⑥
Xu and Xu (2021)	Two arms	Huangkui Capsules: 0.5 g/capsule, 5 capsules/time, Tid	Enalapril: 10 mg, Qd	8 weeks	①②③④⑤

Note: ①: 24 h urine protein; ② Blood creatinine; Serum albumin; Total cholesterol; triglycerides; Adverse reactions.

### 3.3 Assessment of bias risk in included studies

**FIGURE 2:**
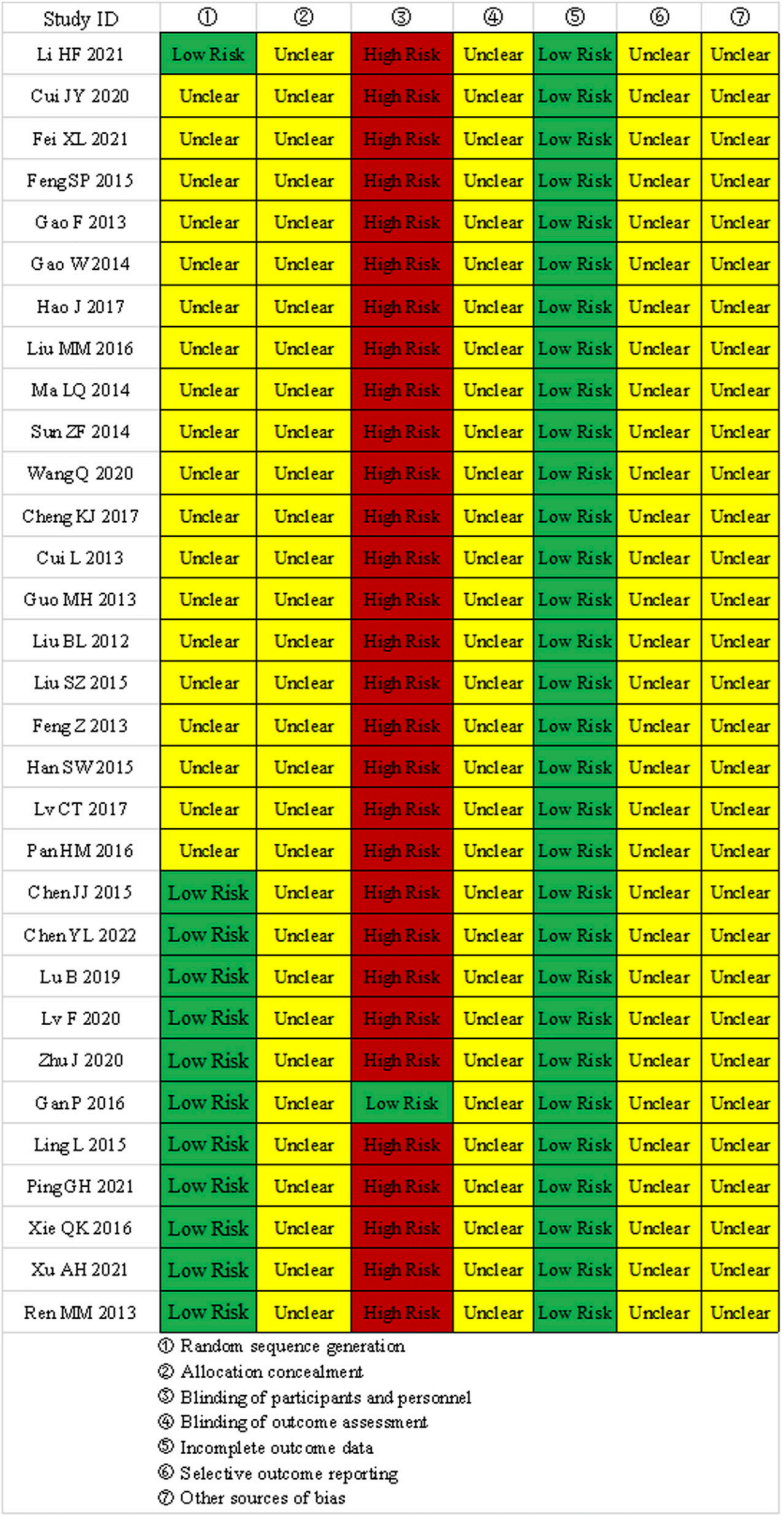
Bias risk evaluation results.

① Random sequence generation: 11 studies used random number tables, 1 study used drawing lots, and 19 studies only mentioned the term “random”; ② Allocation concealment: None of the 31 studies mentioned allocation concealment; ③ Blinding of participants and personnel: 1 study used a placebo, the rest did not implement double-blinding; ④ Blinding of outcome assessment: None of the 31 studies mentioned blinding of outcome assessors; ⑤ Incomplete outcome data: All studies had complete outcome data. ⑥selective reporting and other biases: All studies showed “unclear” selective reporting and other biases. The results of the bias risk assessment are shown in Figure 2.

### 3.4 Paired comparison meta-analysis

#### 3.4.1 Results of a paired comparison meta-analysis of 24-hour urinary protein

The meta-analysis results indicated that HKC + BM, LGT + BM, and SYT + BM were more effective than BM in reducing 24-hour urinary protein levels (*P* < 0.05). However, BLC + BM and WZC + BM did not show a significant difference compared to BM (*P* > 0.05). Descriptive analysis revealed that DHT + BM, HBT + BM, HZC + BM, KXC + BM, MXC + BM, QQC + BM, SKI + BM, and SMI + BM outperformed BM in reducing 24-hour urinary protein (*P* < 0.05), whereas QRG + BM and SFC + BM showed no statistically significant differences compared to BM (*P* > 0.05). Direct comparison data can be found in the Supplementary Table S2. Sensitivity analysis demonstrated that excluding two specific studies (Han et al., 2015; Pan et al., 2016) led to a reversal of the meta-analysis results, indicating that the findings for HKC + BM were not stable (Supplementary Figure S1). Conversely, the sensitivity analysis confirmed that the results for LGT + BM remained stable in reducing 24-hour urinary protein compared to BM (Supplementary Figure S2).

#### 3.4.2 Results of a paired comparison meta-analysis of serum albumin

The meta-analysis results indicated that BLC + BM, HKC + BM, LGT + BM, and SYT + BM were more effective than BM alone in enhancing serum albumin levels (*P* < 0.05). Descriptive analysis revealed that DHT + BM, HBT + BM, HZC + BM, KXC + BM, MXC + BM, QQC + BM, SFC + BM, and SKI + BM outperformed BM in improving serum albumin (*P* < 0.05). However, there was no significant difference between QRG + BM, WZC + BM, and BM (*P* > 0.05). Detailed direct comparisons can be found in the Supplementary Table S3. The sensitivity analysis confirmed that the direct comparison meta-analysis results were consistent and reliable (Supplementary Figures S3, S4).

#### 3.4.3 Results of a paired comparison meta-analysis of serum creatinine

The meta-analysis results indicated that LGT + BM was more effective in improving serum creatinine than BM alone (*P* < 0.05). However, HKC + BM, SYT + BM, and WZC + BM did not show a significant difference compared to BM (*P* > 0.05). Descriptive analysis revealed that BLC + BM, HBT + BM, KXC + BM, and QQC + BM were superior to BM in enhancing serum creatinine levels (*P* < 0.05), whereas HZC + BM, QRG + BM, and SKI + BM did not differ significantly from BM (*P* > 0.05). Direct comparisons are available in the Supplementary Table S4. Sensitivity analysis demonstrated that the meta-analysis results for HKC + BM *versus* BM in improving serum creatinine were consistent (Supplementary Figure S5). Conversely, when studies (Chen et al., 2022; Cui and Li, 2020; Li et al., 2021) were individually excluded, the meta-analysis results for LGT + BM *versus* BM were not stable (Supplementary Figure S6).

#### 3.4.4 Results of a paired comparison meta-analysis of total cholesterol

The meta-analysis results indicated that LGT + BM were more effective than BM alone in improving total cholesterol (*P* < 0.05), whereas HKC + BM did not show any significant difference compared to BM (*P* > 0.05). Descriptive analysis revealed that MXC + BM and QRG + BM outperformed BM in reducing total cholesterol (*P* < 0.05), while HZC + BM and SFC + BM did not present a statistically significant difference when compared to BM (*P* > 0.05). Direct comparisons are provided in the Supplementary Table S5. Sensitivity analysis revealed that excluding the three studies (Feng et al., 2015; Ma et al., 2014; Wang, 2020) respectively caused a reversal in the meta-analysis results. Consequently, the effectiveness of LGT + BM in lowering total cholesterol compared to BM was considered unstable (Supplementary Figure S7).

#### 3.4.5 Results of a paired comparison meta-analysis of triglycerides

The meta-analysis results indicated that LGT combined with BM was more effective than BM alone in improving triglycerides (*P* < 0.05), whereas HKC combined with BM did not show a significant difference compared to BM alone (*P* > 0.05). According to the descriptive analysis, MXC combined with BM surpassed BM alone in reducing triglycerides (*P* < 0.05), but no significant differences were found between HBT combined with BM, QRG combined with BM, SFC combined with BM, and BM alone (*P* > 0.05). Direct comparisons can be found in the Supplementary Table S6. The sensitivity analysis demonstrated that the meta-analysis results for LGT combined with BM in improving triglycerides compared to BM alone were not consistent (Supplementary Figure S8).

#### 3.4.6 Results of a paired comparison meta-analysis of adverse events incidence

The outcomes of direct comparisons indicated no statistically significant differences in adverse event incidence between other interventions and BM, as demonstrated both in the meta-analysis and in descriptive analysis results. Details of these direct comparisons can be found in the Supplementary Table S7. The results of the sensitivity analysis confirmed that the direct comparison meta-analysis results were consistent (Supplementary Figure S9, 10).

#### 3.4.7 Subgroup analysis

Due to the limited number of included studies, it was not feasible to perform a subgroup analysis for each outcome across all interventions. Therefore, this study focused on a subgroup analysis of Leigongteng polysaccharide tablets, categorized by treatment duration (4 months, 6 months, and 12 months). The subgroup analysis results are detailed in the Supplementary Table S8.

Regarding the 24-hour urinary protein outcome, treatment with Leigongteng polysaccharide tablets for 4, 6, and 12 months resulted in a significant reduction in 24-hour urinary protein levels (*P* < 0.05).

As for the serum albumin outcome, treatment with Leigongteng polysaccharide tablets over the same durations led to a significant improvement in serum albumin levels (*P* < 0.05).

Regarding the serum creatinine outcome indicator, after a 4-month intervention with Leigongteng polysaccharide tablets, there was a statistically significant improvement in serum creatinine levels (*P* < 0.05). However, for intervention durations of 6 and 12 months, there was no notable difference in serum creatinine level improvements between Leigongteng polysaccharide tablets and biomedicine (*P* > 0.05).

For the total cholesterol outcome indicator, no statistically significant difference was observed in total cholesterol level improvements between Leigongteng polysaccharide tablets and biomedicine for intervention periods of 6 and 12 months (*P* > 0.05).

Regarding triglycerides, significant improvements were seen in triglyceride levels when treated with Leigongteng polysaccharide tablets for 6 and 12 months, with the differences being statistically significant (*P* < 0.05).

As for the incidence of adverse events, there was no statistically significant difference in adverse event rates between Leigongteng polysaccharide tablets and biomedicine for intervention durations of 4, 6, and 12 months (*P* > 0.05).

### 3.5 Network meta-analysis

#### 3.5.1 Network meta-analysis of the improvement in 24-hour urinary protein

**FIGURE 3:**
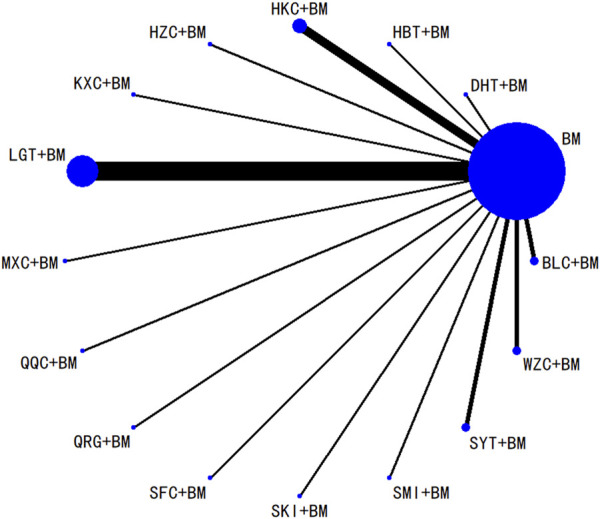
Network plots of degree of improvement of urinary protein in 24 h.

**FIGURE 4:**
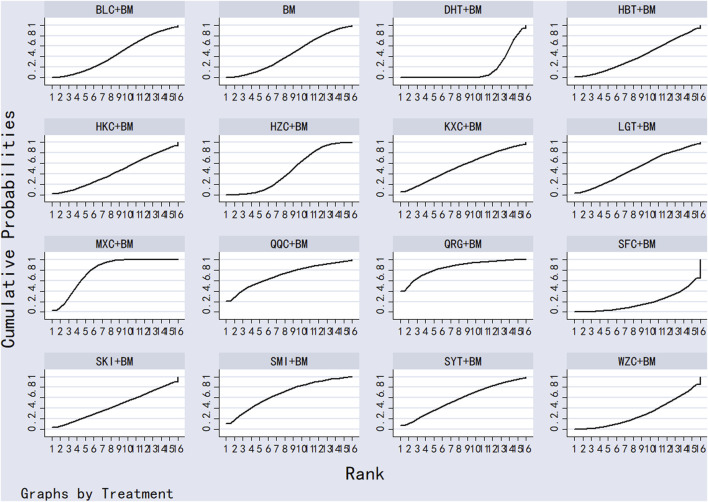
SUCRA plot of degree of improvement of urinary protein in 24 h.

Twenty-nine studies evaluated 24-hour urinary protein levels before and after treatment across 16 different treatment regimens. There were no closed loops among these regimens, and the evidence network is depicted in Figure 3. Given that all comparisons between treatment regimens were indirect, inconsistency tests were unnecessary. Consistency model statistical analysis revealed that QQC + BM and LGT + BM were more effective than BM alone. No significant differences were found among the other treatments, as detailed in Table 3. The SUCRA ranking for improvement in 24-hour urinary protein was as follows: QQC + BM (84.7%) > LGT + BM (78.6%) > MXC + BM (71.4%) > SKI + BM (68.5%) > SMI + BM (57.6%) > HZC + BM (53.7%) > KXC + BM (52.3%) > HKC + BM (46.2%) > SFC + BM (46.2%) > SYT + BM (46.1%) > BLC + BM (45%) > HBT + BM (43.4%) > DHT + BM (41.4%) > WZC + BM (30.3%) > QRG + BM (19.5%) > BM (15.2%), as illustrated in Figure 4.

**TABLE 3:** Network meta analysis results of degree of improvement of urinary protein in 24 h.

QQC+BM	0.43 (−1.15, 2.02)	0.43 (−1.86, 2.72)	0.57 (−1.49, 2.63)	0.83 (−1.25, 2.91)	0.94 (−1.16, 3.04)	0.99 (−1.00, 2.98)	1.13 (−0.52, 2.77)	1.15 (−1.02, 3.32)	1.14 (−0.67, 2.96)	1.16 (−0.68, 3.01)	1.20 (−0.80, 3.20)	1.25 (−0.74, 3.24)	1.54 (−0.43, 3.50)	1.91 (−0.17, 3.99)	1.77 (0.26, 3.28)
−0.43 (−2.02, 1.15)	LGT+BM	−0.00 (−1.78, 1.78)	0.14 (−1.33, 1.61)	0.40 (−1.11, 1.90)	0.51 (−1.03, 2.04)	0.56 (−0.81, 1.93)	0.70 (−0.10, 1.49)	0.72 (−0.91, 2.35)	0.71 (−0.39, 1.82)	0.73 (−0.43, 1.89)	0.77 (−0.62, 2.16)	0.82 (−0.55, 2.19)	1.10 (−0.23, 2.43)	1.48 (−0.02, 2.98)	1.34 (0.87, 1.81)
−0.43 (−2.72, 1.86)	0.00 (−1.78, 1.78)	MXC+BM	0.14 (−2.07, 2.35)	0.40 (−1.84, 2.64)	0.51 (−1.75, 2.77)	0.56 (−1.59, 2.71)	0.70 (−1.14, 2.53)	0.72 (−1.60, 3.04)	0.71 (−1.28, 2.70)	0.73 (−1.29, 2.75)	0.77 (−1.39, 2.93)	0.82 (−1.33, 2.97)	1.11 (−1.02, 3.23)	1.48 (−0.75, 3.71)	1.34 (−0.38, 3.06)
−0.57 (−2.63, 1.49)	−0.14 (−1.61, 1.33)	−0.14 (−2.35, 2.07)	SKI+BM	0.26 (−1.74, 2.26)	0.37 (−1.65, 2.39)	0.42 (−1.48, 2.32)	0.56 (−0.98, 2.10)	0.58 (−1.51, 2.67)	0.57 (−1.14, 2.29)	0.59 (−1.16, 2.34)	0.63 (−1.28, 2.54)	0.68 (−1.22, 2.58)	0.97 (−0.90, 2.84)	1.34 (−0.65, 3.33)	1.20 (−0.19, 2.59)
−0.83 (−2.91, 1.25)	−0.40 (−1.90, 1.11)	−0.40 (−2.64, 1.84)	−0.26 (−2.26, 1.74)	SMI+BM	0.11 (−1.94, 2.16)	0.16 (−1.76, 2.08)	0.30 (−1.27, 1.87)	0.32 (−1.80, 2.44)	0.31 (−1.43, 2.06)	0.33 (−1.45, 2.11)	0.37 (−1.57, 2.31)	0.42 (−1.50, 2.34)	0.71 (−1.19, 2.60)	1.08 (−0.94, 3.10)	0.94 (−0.49, 2.37)
−0.94 (−3.04, 1.16)	−0.51 (−2.04, 1.03)	−0.51 (−2.77, 1.75)	−0.37 (−2.39, 1.65)	−0.11 (−2.16, 1.94)	HZC+BM	0.05 (−1.90, 2.00)	0.19 (−1.41, 1.79)	0.21 (−1.93, 2.35)	0.20 (−1.57, 1.97)	0.22 (−1.58, 2.02)	0.26 (−1.70, 2.22)	0.31 (−1.64, 2.26)	0.60 (−1.33, 2.52)	0.97 (−1.07, 3.01)	0.83 (−0.63, 2.29)
−0.99 (−2.98, 1.00)	−0.56 (−1.93, 0.81)	−0.56 (−2.71, 1.59)	−0.42 (−2.32, 1.48)	−0.16 (−2.08, 1.76)	−0.05 (−2.00, 1.90)	KXC+BM	0.14 (−1.30, 1.58)	0.16 (−1.86, 2.18)	0.15 (−1.48, 1.78)	0.17 (−1.49, 1.84)	0.21 (−1.62, 2.04)	0.26 (−1.56, 2.08)	0.55 (−1.25, 2.34)	0.92 (−1.00, 2.84)	0.78 (−0.50, 2.06)
−1.13 (−2.77, 0.52)	−0.70 (−1.49, 0.10)	−0.70 (−2.53, 1.14)	−0.56 (−2.10, 0.98)	−0.30 (−1.87, 1.27)	−0.19 (−1.79, 1.41)	−0.14 (−1.58, 1.30)	HKC+BM	0.02 (−1.67, 1.71)	0.01 (−1.18, 1.21)	0.03 (−1.21, 1.27)	0.07 (−1.39, 1.53)	0.12 (−1.32, 1.56)	0.41 (−1.00, 1.81)	0.78 (−0.78, 2.35)	0.64 (−0.00, 1.29)
−1.15 (−3.32, 1.02)	−0.72 (−2.35, 0.91)	−0.72 (−3.04, 1.60)	−0.58 (−2.67, 1.51)	−0.32 (−2.44, 1.80)	−0.21 (−2.35, 1.93)	−0.16 (−2.18, 1.86)	−0.02 (−1.71, 1.67)	SFC+BM	−0.01 (−1.86, 1.85)	0.01 (−1.87, 1.90)	0.05 (−1.99, 2.09)	0.10 (−1.92, 2.12)	0.39 (−1.61, 2.38)	0.76 (−1.35, 2.87)	0.62 (−0.94, 2.18)
−1.14 (−2.96, 0.67)	−0.71 (−1.82, 0.39)	−0.71 (−2.70, 1.28)	−0.57 (−2.29, 1.14)	−0.31 (−2.06, 1.43)	−0.20 (−1.97, 1.57)	−0.15 (−1.78, 1.48)	−0.01 (−1.21, 1.18)	0.01 (−1.85, 1.86)	SYT+BM	0.02 (−1.44, 1.47)	0.06 (−1.59, 1.70)	0.11 (−1.52, 1.74)	0.39 (−1.21, 1.99)	0.77 (−0.98, 2.51)	0.63 (−0.38, 1.63)
−1.16 (−3.01, 0.68)	−0.73 (−1.89, 0.43)	−0.73 (−2.75, 1.29)	−0.59 (−2.34, 1.16)	−0.33 (−2.11, 1.45)	−0.22 (−2.02, 1.58)	−0.17 (−1.84, 1.49)	−0.03 (−1.27, 1.21)	−0.01 (−1.90, 1.87)	−0.02 (−1.47, 1.44)	BLC+BM	0.04 (−1.64, 1.72)	0.09 (−1.58, 1.75)	0.37 (−1.26, 2.01)	0.75 (−1.03, 2.52)	0.61 (−0.45, 1.67)
−1.20 (−3.20, 0.80)	−0.77 (−2.16, 0.62)	−0.77 (−2.93, 1.39)	−0.63 (−2.54, 1.28)	−0.37 (−2.31, 1.57)	−0.26 (−2.22, 1.70)	−0.21 (−2.04, 1.62)	−0.07 (−1.53, 1.39)	−0.05 (−2.09, 1.99)	−0.06 (−1.70, 1.59)	−0.04 (−1.72, 1.64)	HBT+BM	0.05 (−1.78, 1.88)	0.34 (−1.47, 2.14)	0.71 (−1.22, 2.64)	0.57 (−0.74, 1.88)
−1.25 (−3.24, 0.74)	−0.82 (−2.19, 0.55)	−0.82 (−2.97, 1.33)	−0.68 (−2.58, 1.22)	−0.42 (−2.34, 1.50)	−0.31 (−2.26, 1.64)	−0.26 (−2.08, 1.56)	−0.12 (−1.56, 1.32)	−0.10 (−2.12, 1.92)	−0.11 (−1.74, 1.52)	−0.09 (−1.75, 1.58)	−0.05 (−1.88, 1.78)	DHT+BM	0.29 (−1.51, 2.08)	0.66 (−1.26, 2.58)	0.52 (−0.77, 1.81)
−1.54 (−3.50, 0.43)	−1.10 (−2.43, 0.23)	−1.11 (−3.23, 1.02)	−0.97 (−2.84, 0.90)	−0.71 (−2.60, 1.19)	−0.60 (−2.52, 1.33)	−0.55 (−2.34, 1.25)	−0.41 (−1.81, 1.00)	−0.39 (−2.38, 1.61)	−0.39 (−1.99, 1.21)	−0.37 (−2.01, 1.26)	−0.34 (−2.14, 1.47)	−0.29 (−2.08, 1.51)	WZC+BM	0.37 (−1.52, 2.27)	0.23 (−1.01, 1.48)
−1.91 (−3.99, 0.17)	−1.48 (−2.98, 0.02)	−1.48 (−3.71, 0.75)	−1.34 (−3.33, 0.65)	−1.08 (−3.10, 0.94)	−0.97 (−3.01, 1.07)	−0.92 (−2.84, 1.00)	−0.78 (−2.35, 0.78)	−0.76 (−2.87, 1.35)	−0.77 (−2.51, 0.98)	−0.75 (−2.52, 1.03)	−0.71 (−2.64, 1.22)	−0.66 (−2.58, 1.26)	−0.37 (−2.27, 1.52)	QRG+BM	−0.14 (−1.57, 1.29)
**−1.77** **(−3.28, −0.26)**	**−1.34** **(−1.81, −0.87)**	−1.34 (−3.06, 0.38)	−1.20 (−2.59, 0.19)	−0.94 (−2.37, 0.49)	−0.83 (−2.29, 0.63)	−0.78 (−2.06, 0.50)	−0.64 (−1.29, 0.00)	−0.62 (−2.18, 0.94)	−0.63 (−1.63, 0.38)	−0.61 (−1.67, 0.45)	−0.57 (−1.88, 0.74)	−0.52 (−1.81, 0.77)	−0.23 (−1.48, 1.01)	0.14 (−1.29, 1.57)	BM

Note: The above data are confidence intervals, and bold characters indicate statistically significant differences.

#### 3.5.2 Network analysis of serum albumin improvement

**FIGURE 5:**
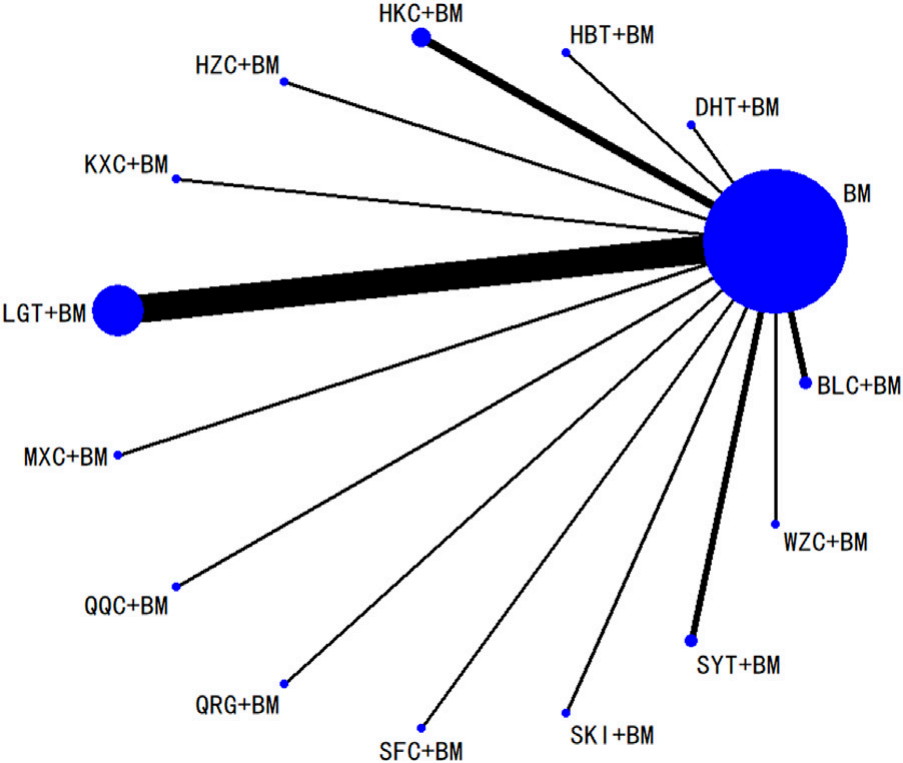
Network plots of degree of improvement of serum albumin.

**FIGURE 6:**
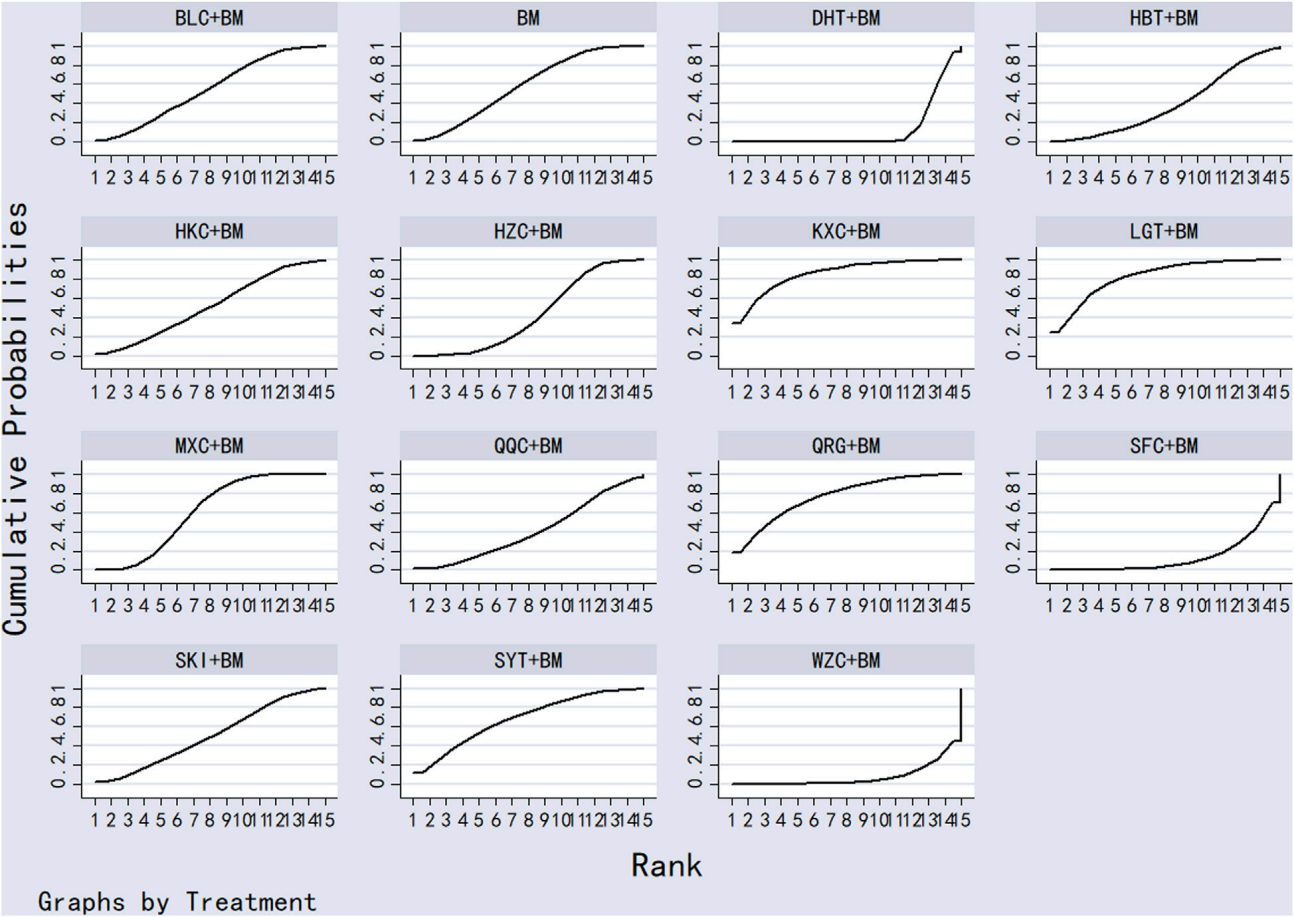
SUCRA plot of degree of improvement of serum albumin.

Twenty-six studies examined serum albumin across 15 treatment regimens, with no closed loops present between the various regimens, as depicted in Figure 5. All pairwise comparisons between treatment regimens were based on indirect comparisons, rendering inconsistency tests unnecessary. Consistency model statistical analysis revealed that HZC + BM outperformed QRG + BM, BM, and WZC + BM; KXC + BM was superior to QRG + BM, BM, and WZC + BM; QQC + BM outshone QRG + BM, BM, and WZC + BM; SKI + BM was better than BM; LGT + BM surpassed BM and WZC + BM; while SYT + BM, HKC + BM, and BLC + BM all showed superiority over BM. No significant differences were found among the other treatment regimens. More information is available in Table 4. The SUCRA ranking for serum albumin improvement was as follows: HZC + BM (86%) > KXC + BM (83%) > QQC + BM (76.6%) > SKI + BM (68.9%) > LGT + BM (61%) > SYT + BM (59%) > BLC + BM (55.7%) > HBT + BM (51.9%) > SFC + BM (50.7%) > HKC + BM (42.3%) > MXC + BM (41.1%) > DHT + BM (39.5%) > QRG + BM (13.7%) > BM (12.7%) > WZC + BM (8.1%), with the SUCRA ranking illustrated in Figure 6.

**TABLE 4:** Network meta analysis results of degree of improvement of serum albumin.

HZC+BM	−0.61 (−7.76, 6.54)	−1.35 (−8.78, 6.08)	−2.23 (−10.04, 5.58)	−3.34 (−8.91, 2.23)	−3.48 (−9.94, 2.97)	−3.75 (−10.49, 3.00)	−4.12 (−11.27, 3.03)	−4.23 (−11.54, 3.08)	−4.93 (−10.93, 1.08)	−5.29 (−12.82, 2.24)	−5.38 (−12.48, 1.72)	−8.81 (−16.33, −1.29)	−8.23 (−13.54, −2.92)	−10.23 (−18.07, −2.39)
0.61 (−6.54, 7.76)	KXC+BM	−0.74 (−7.81, 6.33)	−1.62 (−9.09, 5.85)	−2.73 (−7.82, 2.36)	−2.87 (−8.92, 3.17)	−3.14 (−9.48, 3.21)	−3.51 (−10.28, 3.26)	−3.62 (−10.57, 3.33)	−4.32 (−9.87, 1.24)	−4.68 (−11.86, 2.50)	−4.77 (−11.49, 1.95)	−8.20 (−15.37, −1.03)	−7.62 (−12.41, −2.83)	−9.62 (−17.12, −2.12)
1.35 (−6.08, 8.78)	0.74 (−6.33, 7.81)	QQC+BM	−0.88 (−8.61, 6.85)	−1.99 (−7.46, 3.48)	−2.13 (−8.50, 4.24)	−2.40 (−9.06, 4.27)	−2.77 (−9.84, 4.30)	−2.88 (−10.12, 4.36)	−3.58 (−9.49, 2.33)	−3.94 (−11.40, 3.52)	−4.03 (−11.05, 2.99)	−7.46 (−14.90, −0.02)	−6.88 (−12.08, −1.68)	−8.88 (−16.65, −1.11)
2.23 (−5.58, 10.04)	1.62 (−5.85, 9.09)	0.88 (−6.85, 8.61)	SKI+BM	−1.11 (−7.08, 4.86)	−1.25 (−8.06, 5.55)	−1.52 (−8.59, 5.56)	−1.89 (−9.35, 5.57)	−2.00 (−9.62, 5.62)	−2.70 (−9.07, 3.68)	−3.06 (−10.89, 4.77)	−3.15 (−10.56, 4.26)	−6.58 (−14.40, 1.24)	−6.00 (−11.73, −0.27)	−8.00 (−16.13, 0.13)
3.34 (−2.23, 8.91)	2.73 (−2.36, 7.82)	1.99 (−3.48, 7.46)	1.11 (−4.86, 7.08)	LGT+BM	−0.14 (−4.19, 3.90)	−0.41 (−4.90, 4.08)	−0.78 (−5.85, 4.29)	−0.89 (−6.20, 4.42)	−1.59 (−4.86, 1.69)	−1.95 (−7.56, 3.66)	−2.04 (−7.04, 2.97)	−5.47 (−11.06, 0.12)	−4.89 (−6.58, −3.20)	−6.89 (−12.90, −0.88)
3.48 (−2.97, 9.94)	2.87 (−3.17, 8.92)	2.13 (−4.24, 8.50)	1.25 (−5.55, 8.06)	0.14 (−3.90, 4.19)	SYT+BM	−0.26 (−5.82, 5.29)	−0.64 (−6.67, 5.40)	−0.75 (−6.98, 5.49)	−1.44 (−6.07, 3.18)	−1.81 (−8.29, 4.68)	−1.89 (−7.87, 4.08)	−5.33 (−11.80, 1.15)	−4.75 (−8.42, −1.07)	−6.75 (−13.59, 0.09)
3.75 (−3.00, 10.49)	3.14 (−3.21, 9.48)	2.40 (−4.27, 9.06)	1.52 (−5.56, 8.59)	0.41 (−4.08, 4.90)	0.26 (−5.29, 5.82)	BLC+BM	−0.37 (−6.71, 5.96)	−0.48 (−7.01, 6.04)	−1.18 (−6.20, 3.84)	−1.54 (−8.32, 5.23)	−1.63 (−7.92, 4.65)	−5.06 (−11.82, 1.69)	−4.48 (−8.65, −0.32)	−6.48 (−13.60, 0.63)
4.12 (−3.03, 11.27)	3.51 (−3.26, 10.28)	2.77 (−4.30, 9.84)	1.89 (−5.57, 9.35)	0.78 (−4.29, 5.85)	0.64 (−5.40, 6.67)	0.37 (−5.96, 6.71)	HBT+BM	−0.11 (−7.05, 6.83)	−0.81 (−6.35, 4.74)	−1.17 (−8.34, 6.00)	−1.26 (−7.97, 5.45)	−4.69 (−11.85, 2.47)	−4.11 (−8.89, 0.67)	−6.11 (−13.60, 1.38)
4.23 (−3.08, 11.54)	3.62 (−3.33, 10.57)	2.88 (−4.36, 10.12)	2.00 (−5.62, 9.62)	0.89 (−4.42, 6.20)	0.75 (−5.49, 6.98)	0.48 (−6.04, 7.01)	0.11 (−6.83, 7.05)	SFC+BM	−0.70 (−6.46, 5.06)	−1.06 (−8.40, 6.28)	−1.15 (−8.04, 5.74)	−4.58 (−11.91, 2.75)	−4.00 (−9.03, 1.03)	−6.00 (−13.65, 1.65)
4.93 (−1.08, 10.93)	4.32 (−1.24, 9.87)	3.58 (−2.33, 9.49)	2.70 (−3.68, 9.07)	1.59 (−1.69, 4.86)	1.44 (−3.18, 6.07)	1.18 (−3.84, 6.20)	0.81 (−4.74, 6.35)	0.70 (−5.06, 6.46)	HKC+BM	−0.36 (−6.40, 5.67)	−0.45 (−5.94, 5.03)	−3.88 (−9.90, 2.14)	−3.30 (−6.11, −0.50)	−5.30 (−11.72, 1.11)
5.29 (−2.24, 12.82)	4.68 (−2.50, 11.86)	3.94 (−3.52, 11.40)	3.06 (−4.77, 10.89)	1.95 (−3.66, 7.56)	1.81 (−4.68, 8.29)	1.54 (−5.23, 8.32)	1.17 (−6.00, 8.34)	1.06 (−6.28, 8.40)	0.36 (−5.67, 6.40)	MXC+BM	−0.09 (−7.21, 7.04)	−3.52 (−11.07, 4.03)	−2.94 (−8.28, 2.40)	−4.94 (−12.80, 2.92)
5.38 (−1.72, 12.48)	4.77 (−1.95, 11.49)	4.03 (−2.99, 11.05)	3.15 (−4.26, 10.56)	2.04 (−2.97, 7.04)	1.89 (−4.08, 7.87)	1.63 (−4.65, 7.92)	1.26 (−5.45, 7.97)	1.15 (−5.74, 8.04)	0.45 (−5.03, 5.94)	0.09 (−7.04, 7.21)	DHT+BM	−3.43 (−10.54, 3.68)	−2.85 (−7.56, 1.86)	−4.85 (−12.30, 2.60)
**8.81** **(1.29, 16.33)**	**8.20** **(1.03, 15.37)**	**7.46** **(0.02, 14.90)**	6.58 (−1.24, 14.40)	5.47 (−0.12, 11.06)	5.33 (−1.15, 11.80)	5.06 (−1.69, 11.82)	4.69 (−2.47, 11.85)	4.58 (−2.75, 11.91)	3.88 (−2.14, 9.90)	3.52 (−4.03, 11.07)	3.43 (−3.68, 10.54)	QRG+BM	0.58 (−4.75, 5.91)	−1.42 (−9.27, 6.43)
**8.23** **(2.92, 13.54)**	**7.62** **(2.83, 12.41)**	**6.88** **(1.68, 12.08)**	**6.00** **(0.27, 11.73)**	**4.89** **(3.20, 6.58)**	**4.75** **(1.07, 8.42)**	**4.48** **(0.32, 8.65)**	4.11 (−0.67, 8.89)	4.00 (−1.03, 9.03)	**3.30** **(0.50, 6.11)**	2.94 (−2.40, 8.28)	2.85 (−1.86, 7.56)	−0.58 (−5.91, 4.75)	BM	−2.00 (−7.77, 3.77)
**10.23** **(2.39, 18.07)**	**9.62** **(2.12, 17.12)**	**8.88** **(1.11, 16.65)**	8.00 (−0.13, 16.13)	**6.89** **(0.88, 12.90)**	6.75 (−0.09, 13.59)	6.48 (−0.63, 13.60)	6.11 (−1.38, 13.60)	6.00 (−1.65, 13.65)	5.30 (−1.11, 11.72)	4.94 (−2.92, 12.80)	4.85 (−2.60, 12.30)	1.42 (−6.43, 9.27)	2.00 (−3.77, 7.77)	WZC+BM

The above data are confidence intervals, and bold characters indicate statistically significant differences.

#### 3.5.3 Network analysis of serum creatinine improvement

**FIGURE 7:**
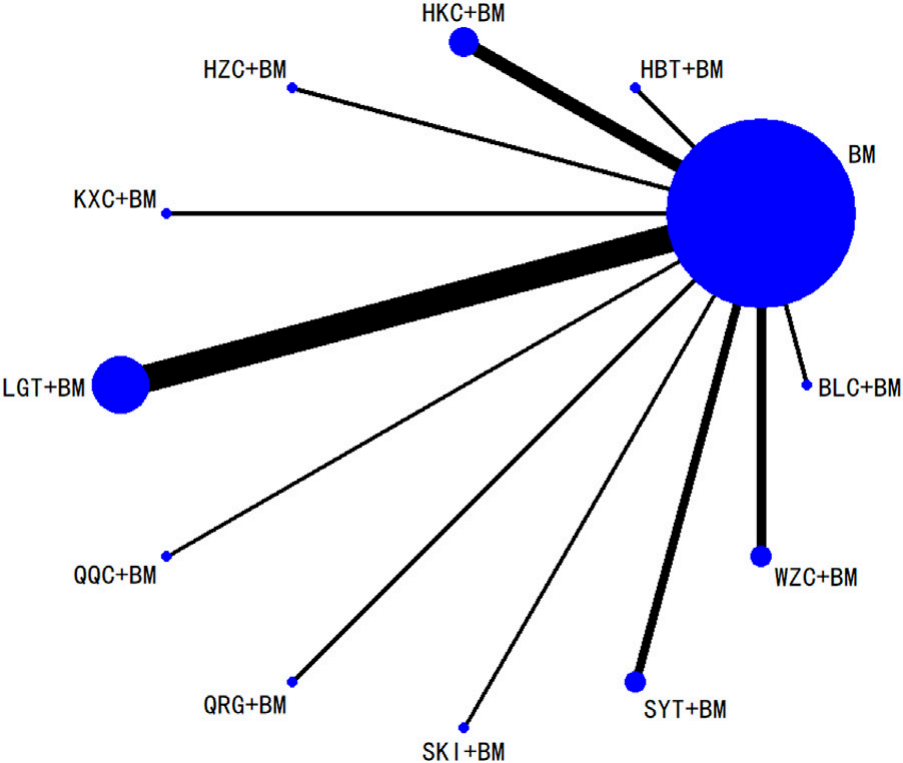
Network plots of degree of improvement of blood creatinine.

**FIGURE 8:**
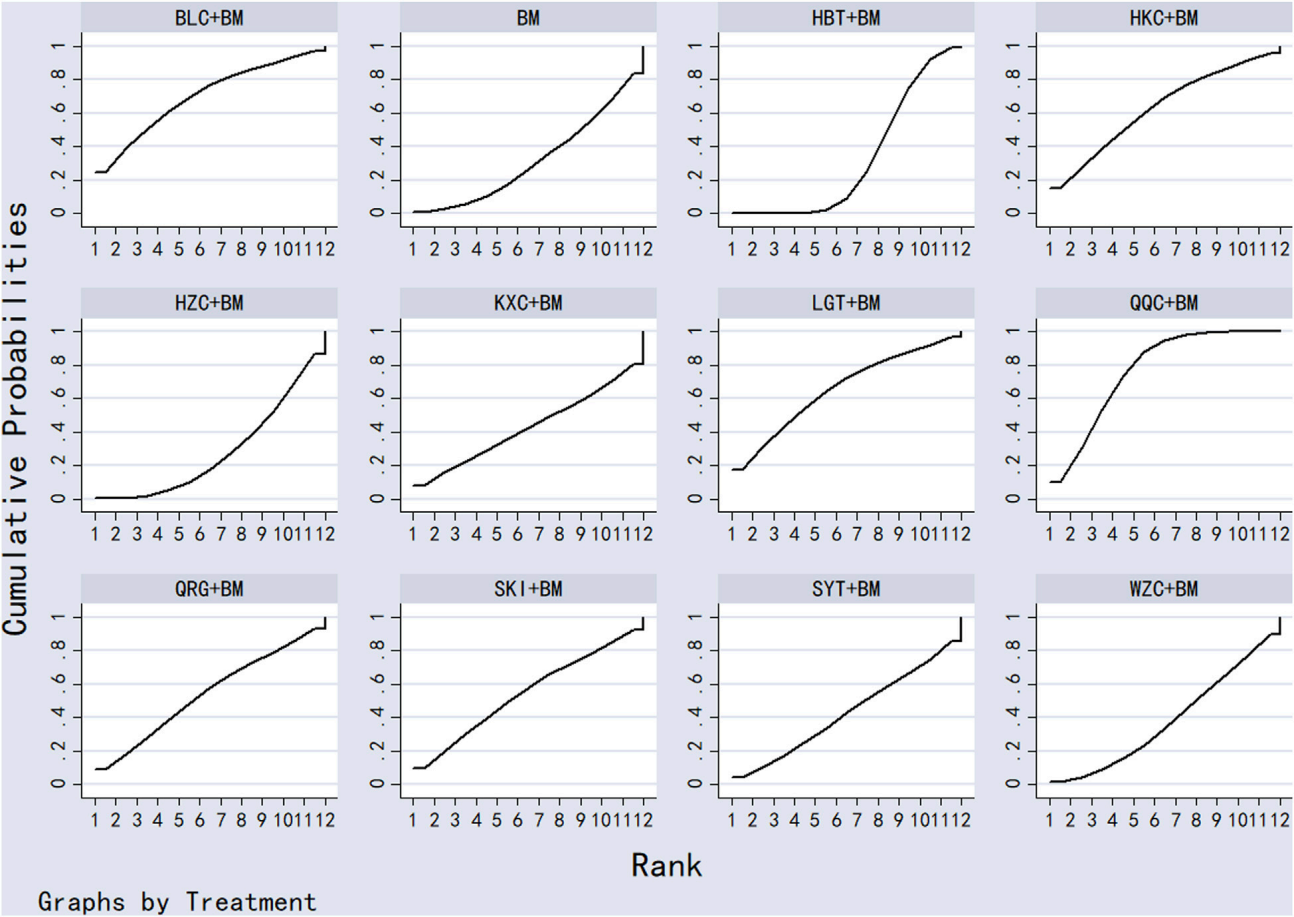
SUCRA plot of degree of improvement of blood creatinine.

Twenty studies investigated serum creatinine across 12 treatment regimens, also without closed loops among the regimens. The evidence network diagram is shown in Figure 7. Since all pairwise comparisons between the treatments were indirect, inconsistency tests were unnecessary. The statistical analysis using a consistency model demonstrated that LGT + BM was superior to BM. No significant differences were found among other treatment regimens, as detailed in Table 5. The SUCRA ranking for serum creatinine improvement was as follows: LGT + BM (77.2%) > BLC + BM (70.3%) > KXC + BM (64.7%) > HBT + BM (62.8%) > QRG + BM (55%) > QQC + BM (54.6%) > HZC + BM (43.2%) > SKI + BM (42.2%) > WZC + BM (38%) > SYT + BM (32.4%) > BM (31.9%) > HKC + BM (27.6%), with the SUCRA ranking graph shown in Figure 8.

**TABLE 5:** Network meta analysis results of degree of improvement of serum creatinine.

LGT+BM	0.20 (−23.60, 23.99)	2.21 (−19.80, 24.23)	3.29 (−18.50, 25.09)	5.77 (−16.91, 28.45)	6.22 (−15.46, 27.90)	10.02 (−16.50, 36.55)	10.19 (−12.01, 32.40)	11.19 (−6.31, 28.70)	13.13 (−4.80, 31.06)	12.19 (3.49, 20.90)	13.75 (−0.85, 28.35)
−0.20 (−23.99, 23.60)	BLC+BM	2.02 (−27.97, 32.00)	3.10 (−26.72, 32.92)	5.58 (−24.90, 36.05)	6.03 (−23.71, 35.77)	9.83 (−23.61, 43.26)	10.00 (−20.13, 40.12)	11.00 (−15.85, 37.85)	12.93 (−14.19, 40.06)	12.00 (−10.14, 34.14)	13.56 (−11.49, 38.61)
−2.21 (−24.23, 19.80)	−2.02 (−32.00, 27.97)	KXC+BM	1.08 (−27.35, 29.51)	3.56 (−25.55, 32.67)	4.01 (−24.33, 32.35)	7.81 (−24.39, 40.01)	7.98 (−20.77, 36.72)	8.98 (−16.31, 34.27)	10.92 (−14.67, 36.50)	9.98 (−10.24, 30.20)	11.54 (−11.84, 34.91)
−3.29 (−25.09, 18.50)	−3.10 (−32.92, 26.72)	−1.08 (−29.51, 27.35)	HBT+BM	2.48 (−26.47, 31.43)	2.93 (−25.24, 31.10)	6.73 (−25.32, 38.78)	6.90 (−21.67, 35.47)	7.90 (−17.20, 33.00)	9.84 (−15.55, 35.23)	8.90 (−11.08, 28.88)	10.46 (−12.71, 33.62)
−5.77 (−28.45, 16.91)	−5.58 (−36.05, 24.90)	−3.56 (−32.67, 25.55)	−2.48 (−31.43, 26.47)	QRG+BM	0.45 (−28.41, 29.31)	4.25 (−28.40, 36.90)	4.42 (−24.84, 33.68)	5.42 (−20.45, 31.29)	7.36 (−18.80, 33.51)	6.42 (−14.52, 27.36)	7.98 (−16.02, 31.98)
−6.22 (−27.90, 15.46)	−6.03 (−35.77, 23.71)	−4.01 (−32.35, 24.33)	−2.93 (−31.10, 25.24)	−0.45 (−29.31, 28.41)	QQC+BM	3.80 (−28.17, 35.77)	3.97 (−24.52, 32.46)	4.97 (−20.03, 29.97)	6.91 (−18.38, 32.20)	5.97 (−13.88, 25.83)	7.53 (−15.53, 30.58)
−10.02 (−36.55, 16.50)	−9.83 (−43.26, 23.61)	−7.81 (−40.01, 24.39)	−6.73 (−38.78, 25.32)	−4.25 (−36.90, 28.40)	−3.80 (−35.77, 28.17)	HZC+BM	0.17 (−32.16, 32.50)	1.17 (−28.13, 30.47)	3.11 (−26.44, 32.66)	2.17 (−22.88, 27.22)	3.73 (−23.93, 31.39)
−10.19 (−32.40, 12.01)	−10.00 (−40.12, 20.13)	−7.98 (−36.72, 20.77)	−6.90 (−35.47, 21.67)	−4.42 (−33.68, 24.84)	−3.97 (−32.46, 24.52)	−0.17 (−32.50, 32.16)	SKI+BM	1.00 (−24.46, 26.46)	2.94 (−22.81, 28.68)	2.00 (−18.43, 22.43)	3.56 (−19.99, 27.11)
−11.19 (−28.70, 6.31)	−11.00 (−37.85, 15.85)	−8.98 (−34.27, 16.31)	−7.90 (−33.00, 17.20)	−5.42 (−31.29, 20.45)	−4.97 (−29.97, 20.03)	−1.17 (−30.47, 28.13)	−1.00 (−26.46, 24.46)	WZC+BM	1.94 (−19.88, 23.76)	1.00 (−14.19, 16.19)	2.56 (−16.63, 21.74)
−13.13 (−31.06, 4.80)	−12.93 (−40.06, 14.19)	−10.92 (−36.50, 14.67)	−9.84 (−35.23, 15.55)	−7.36 (−33.51, 18.80)	−6.91 (−32.20, 18.38)	−3.11 (−32.66, 26.44)	−2.94 (−28.68, 22.81)	−1.94 (−23.76, 19.88)	SYT+BM	−0.94 (−16.60, 14.73)	0.62 (−18.94, 20.19)
**−12.19** **(−20.90, −3.49)**	−12.00 (−34.14, 10.14)	−9.98 (−30.20, 10.24)	−8.90 (−28.88, 11.08)	−6.42 (−27.36, 14.52)	−5.97 (−25.83, 13.88)	−2.17 (−27.22, 22.88)	−2.00 (−22.43, 18.43)	−1.00 (−16.19, 14.19)	0.94 (−14.73, 16.60)	BM	1.56 (−10.16, 13.28)
−13.75 (−28.35, 0.85)	−13.56 (−38.61, 11.49)	−11.54 (−34.91, 11.84)	−10.46 (−33.62, 12.71)	−7.98 (−31.98, 16.02)	−7.53 (−30.58, 15.53)	−3.73 (−31.39, 23.93)	−3.56 (−27.11, 19.99)	−2.56 (−21.74, 16.63)	−0.62 (−20.19, 18.94)	−1.56 (−13.28, 10.16)	HKC+BM

Note: The above data are confidence intervals, and bold characters indicate statistically significant differences.

#### 3.5.4 Network analysis of total cholesterol improvement

**FIGURE 9:**
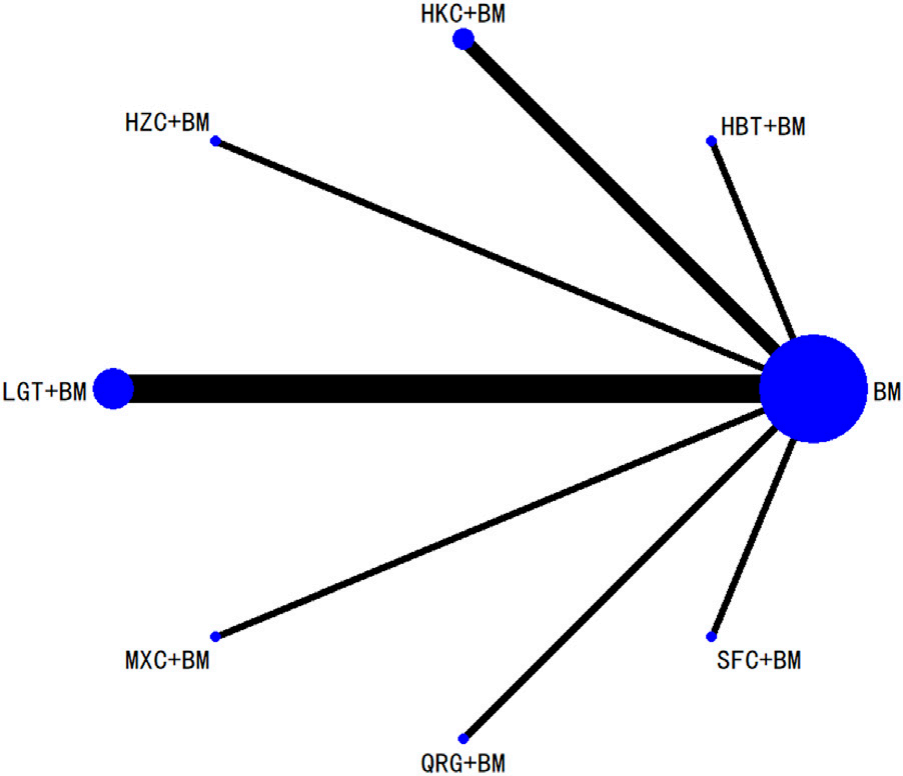
Network plots of degree of improvement in total cholesterol.

**FIGURE 10:**
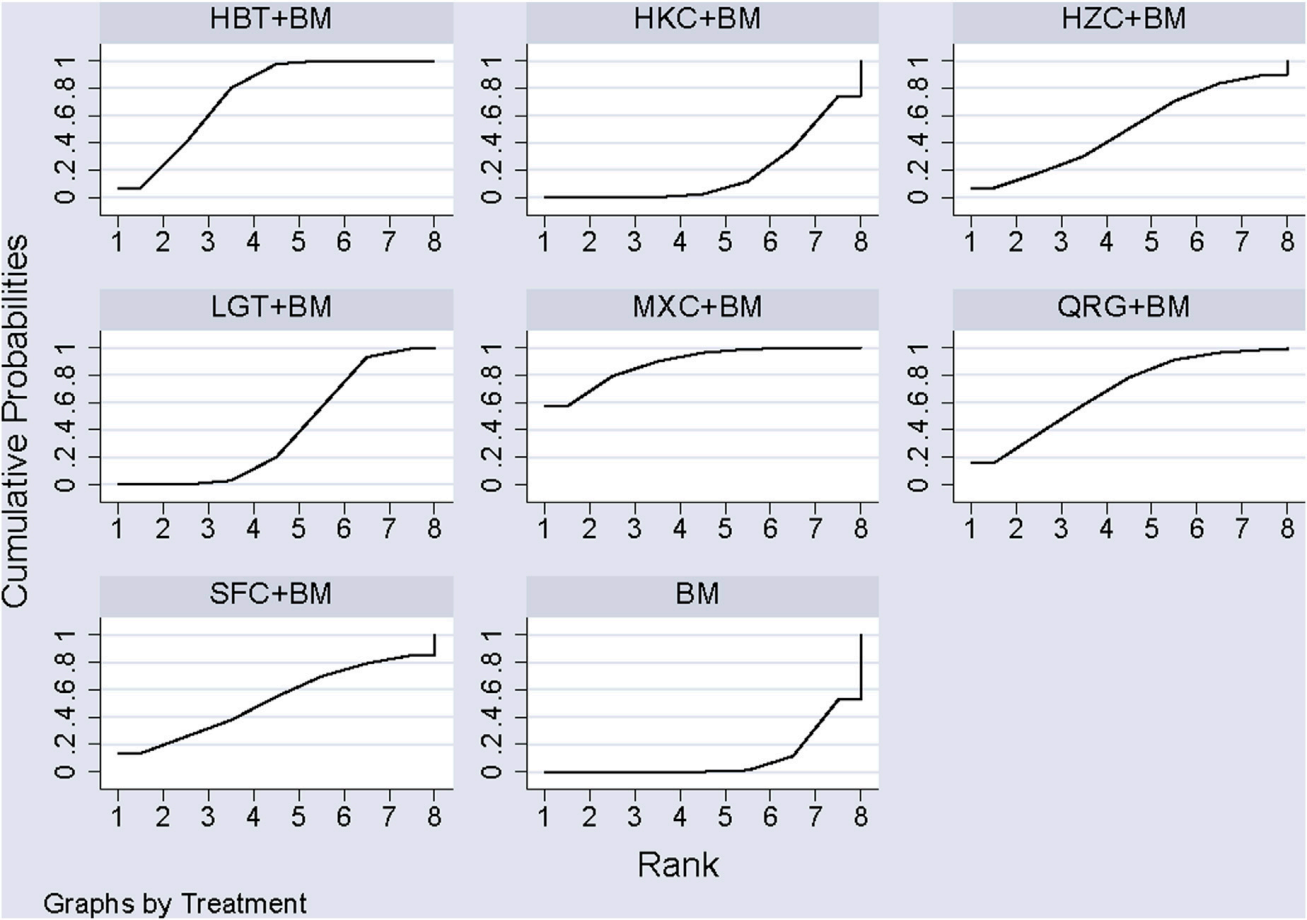
SUCRA plot of degree of improvement in total cholesterol.

A total of 11 studies examined total cholesterol levels across 8 treatment regimens. There were no closed loops among these regimens, as illustrated in the evidence network graph in Figure 9. Since all comparisons between treatment regimens were indirect, inconsistency tests were unnecessary. Consistency model statistical analysis showed that MXC + BM outperformed LGT + BM, HKC + BM, and BM; HBT + BM was more effective than LGT + BM, HKC + BM, and BM; and both QRG + BM and LGT + BM were better than BM. No significant differences were found among the other treatment regimens (see Table 6). The SUCRA ranking for total cholesterol improvement was as follows: MXC + BM (89%) > HBT + BM (74.7%) > QRG + BM (68.2%) > SFC + BM (52.9%) > HZC + BM (49.5%) > LGT + BM (38.8%) > HKC + BM (17.5%) > BM (9.3%), with the SUCRA ranking graph displayed in Figure 10.

**TABLE 6:** Network meta analysis results of degree of improvement in total cholesterol.

MXC+BM	0.32 (−0.45, 1.09)	0.39 (−0.70, 1.48)	0.61 (−0.78, 2.00)	0.67 (−0.52, 1.86)	0.86 (0.05, 1.67)	1.12 (0.29, 1.96)	1.20 (0.44, 1.96)
−0.32 (−1.09, 0.45)	HBT+BM	0.07 (−0.72, 0.86)	0.29 (−0.89, 1.47)	0.35 (−0.57, 1.27)	0.54 (0.23, 0.85)	0.80 (0.44, 1.17)	0.88 (0.75, 1.01)
−0.39 (−1.48, 0.70)	−0.07 (−0.86, 0.72)	QRG+BM	0.22 (−1.18, 1.62)	0.28 (−0.92, 1.48)	0.47 (−0.36, 1.29)	0.73 (−0.12, 1.58)	0.81 (0.03, 1.59)
−0.61 (−2.00, 0.78)	−0.29 (−1.47, 0.89)	−0.22 (−1.62, 1.18)	SFC+BM	0.06 (−1.42, 1.54)	0.25 (−0.95, 1.45)	0.51 (−0.71, 1.73)	0.59 (−0.58, 1.76)
−0.67 (−1.86, 0.52)	−0.35 (−1.27, 0.57)	−0.28 (−1.48, 0.92)	−0.06 (−1.54, 1.42)	HZC+BM	0.19 (−0.77, 1.14)	0.45 (−0.52, 1.43)	0.53 (−0.38, 1.44)
**−0.86** **(−1.67, −0.05)**	**−0.54** **(−0.85, −0.23)**	−0.47 (−1.29, 0.36)	−0.25 (−1.45, 0.95)	−0.19 (−1.14, 0.77)	LGT+BM	0.26 (−0.18, 0.71)	0.34 (0.06, 0.62)
**−1.12** **(−1.96, −0.29)**	**−0.80** **(−1.17, −0.44)**	−0.73 (−1.58, 0.12)	−0.51 (−1.73, 0.71)	−0.45 (−1.43, 0.52)	−0.26 (−0.71, 0.18)	HKC+BM	0.08 (−0.27, 0.42)
**−1.20** **(−1.96, −0.44)**	**−0.88** **(−1.01, −0.75)**	**−0.81** **(−1.59, −0.03)**	−0.59 (−1.76, 0.58)	−0.53 (−1.44, 0.38)	**−0.34** **(−0.62, −0.06)**	−0.08 (−0.42, 0.27)	BM

Note: The above data are confidence intervals, and bold characters indicate statistically significant differences.

#### 3.5.5 Network analysis of triglycerides improvement

**FIGURE 11:**
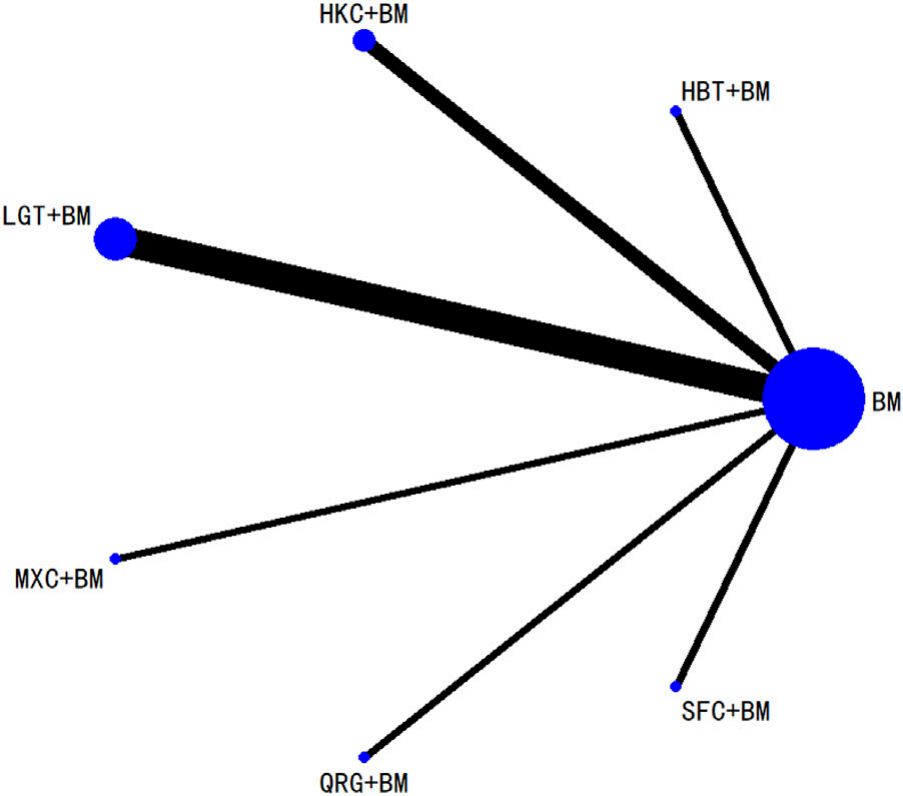
Network plots of degree of improvement of triglycerides.

**FIGURE 12:**
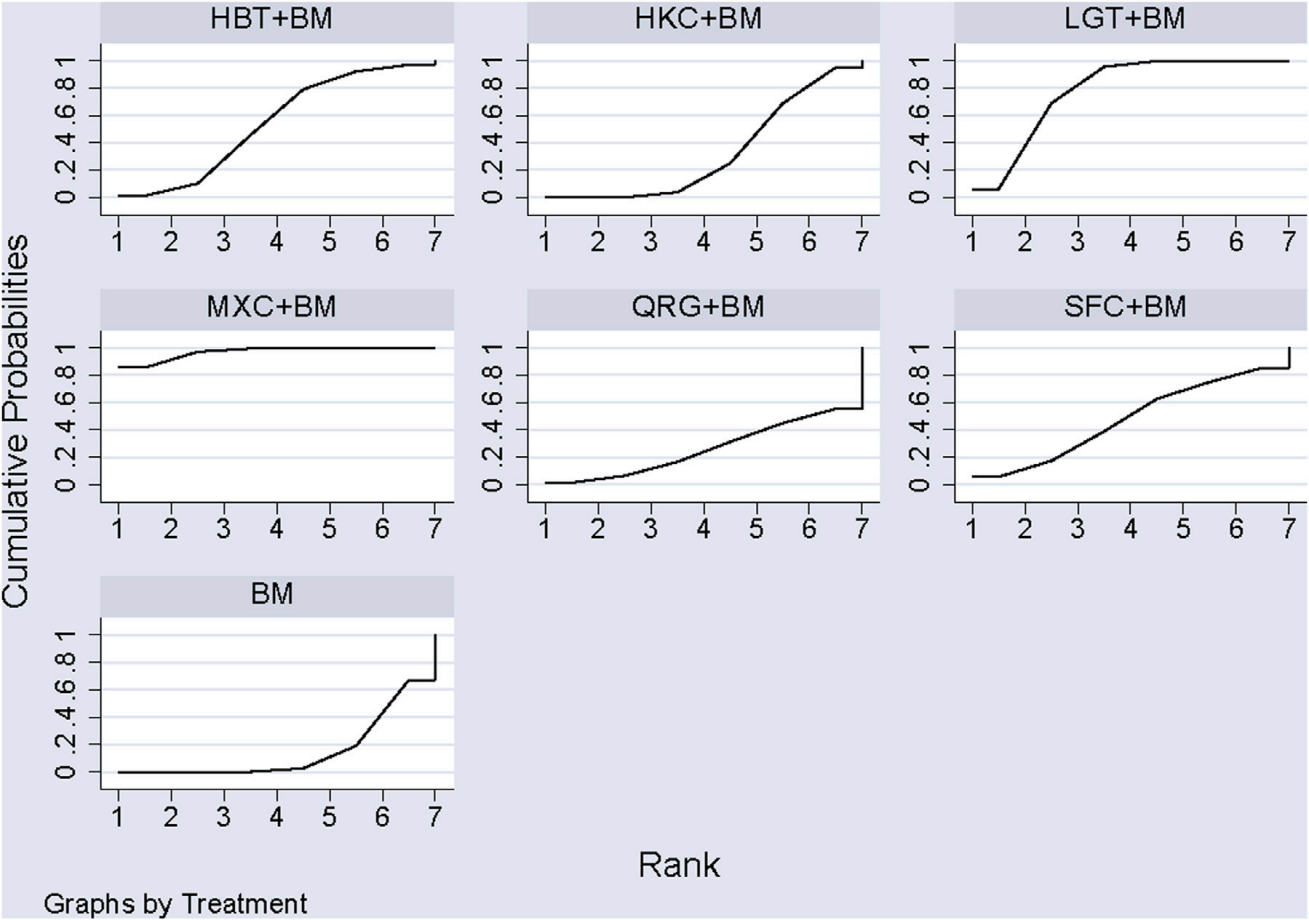
SUCRA plot of degree of triglyceride improvement.

Ten studies examined triglycerides across seven treatment regimens, with no closed loops among them, as depicted in the evidence network graph in Figure 11. Since all pairwise comparisons between treatment regimens were indirect, inconsistency tests were unnecessary. Consistency model statistical analysis revealed that MXC + BM was more effective than HBT + BM, QRG + BM, HKC + BM, and BM, while LGT + BM outperformed HKC + BM and BM, with no significant differences among the other regimens, as detailed in Table 7. The SUCRA ranking for triglyceride improvement was: MXC + BM (97%) > LGT + BM (77.9%) > HBT + BM (54.2%) > SFC + BM (47.7%) > HKC + BM (32%) > QRG + BM (26.8%) > BM (14.3%), illustrated in the SUCRA ranking graph in Figure 12.

**TABLE 7:** Network meta analysis results of degree of improvement of triglycerides.

MXC+BM	0.26 (−0.09, 0.62)	0.50 (0.00, 1.00)	0.56 (−0.14, 1.26)	0.71 (0.36, 1.07)	0.78 (0.02, 1.54)	0.80 (0.48, 1.12)
−0.26 (−0.62, 0.09)	LGT+BM	0.24 (−0.17, 0.65)	0.30 (−0.34, 0.94)	0.45 (0.25, 0.66)	0.52 (−0.19, 1.22)	0.54 (0.39, 0.69)
**−0.50** **(−1.00, −0.00)**	−0.24 (−0.65, 0.17)	HBT+BM	0.06 (−0.67, 0.79)	0.21 (−0.19, 0.62)	0.28 (−0.51, 1.07)	0.30 (−0.08, 0.68)
−0.56 (−1.26, 0.14)	−0.30 (−0.94, 0.34)	−0.06 (−0.79, 0.67)	SFC+BM	0.15 (−0.48, 0.79)	0.22 (−0.71, 1.15)	0.24 (−0.38, 0.86)
**−0.71** **(−1.07, −0.36)**	**−0.45** **(−0.66, −0.25)**	−0.21 (−0.62, 0.19)	−0.15 (−0.79, 0.48)	HKC+BM	0.07 (−0.64, 0.77)	0.09 (−0.06, 0.23)
**−0.78** **(−1.54, −0.02)**	−0.52 (−1.22, 0.19)	−0.28 (−1.07, 0.51)	−0.22 (−1.15, 0.71)	−0.07 (−0.77, 0.64)	QRG+BM	0.02 (−0.67, 0.71)
**−0.80** **(−1.12, −0.48)**	**−0.54** **(−0.69, −0.39)**	−0.30 (−0.68, 0.08)	−0.24 (−0.86, 0.38)	−0.09 (−0.23, 0.06)	−0.02 (−0.71, 0.67)	BM

Note: The above data are confidence intervals, and bold characters indicate statistically significant differences.

#### 3.5.6 Network analysis of adverse events incidence

**FIGURE 13:**
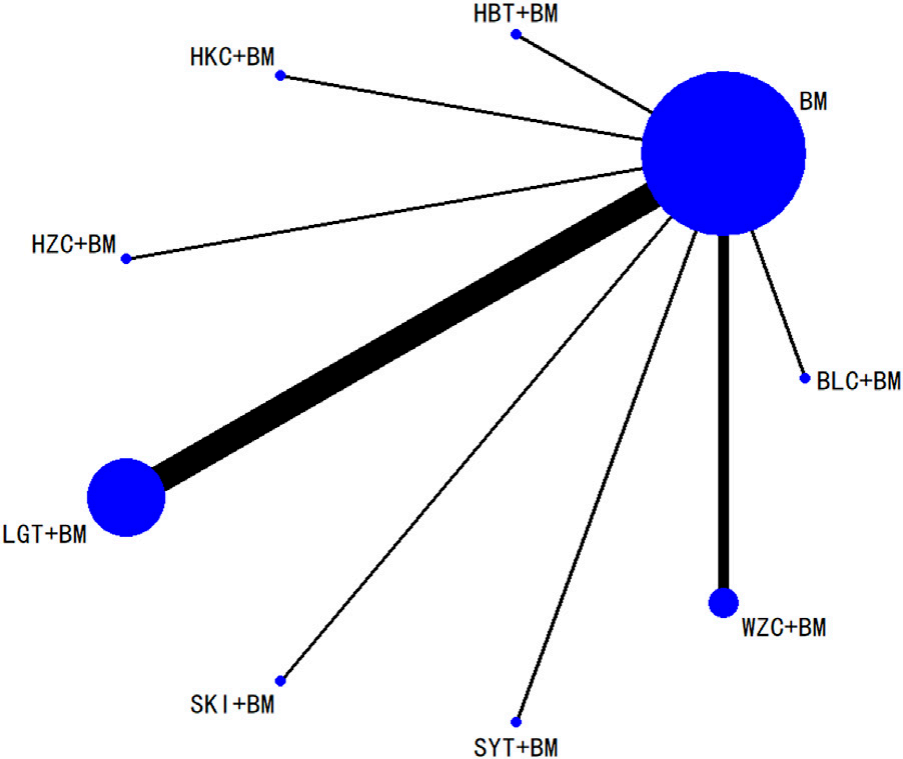
Network plots of the incidence of adverse reactions.

**FIGURE 14:**
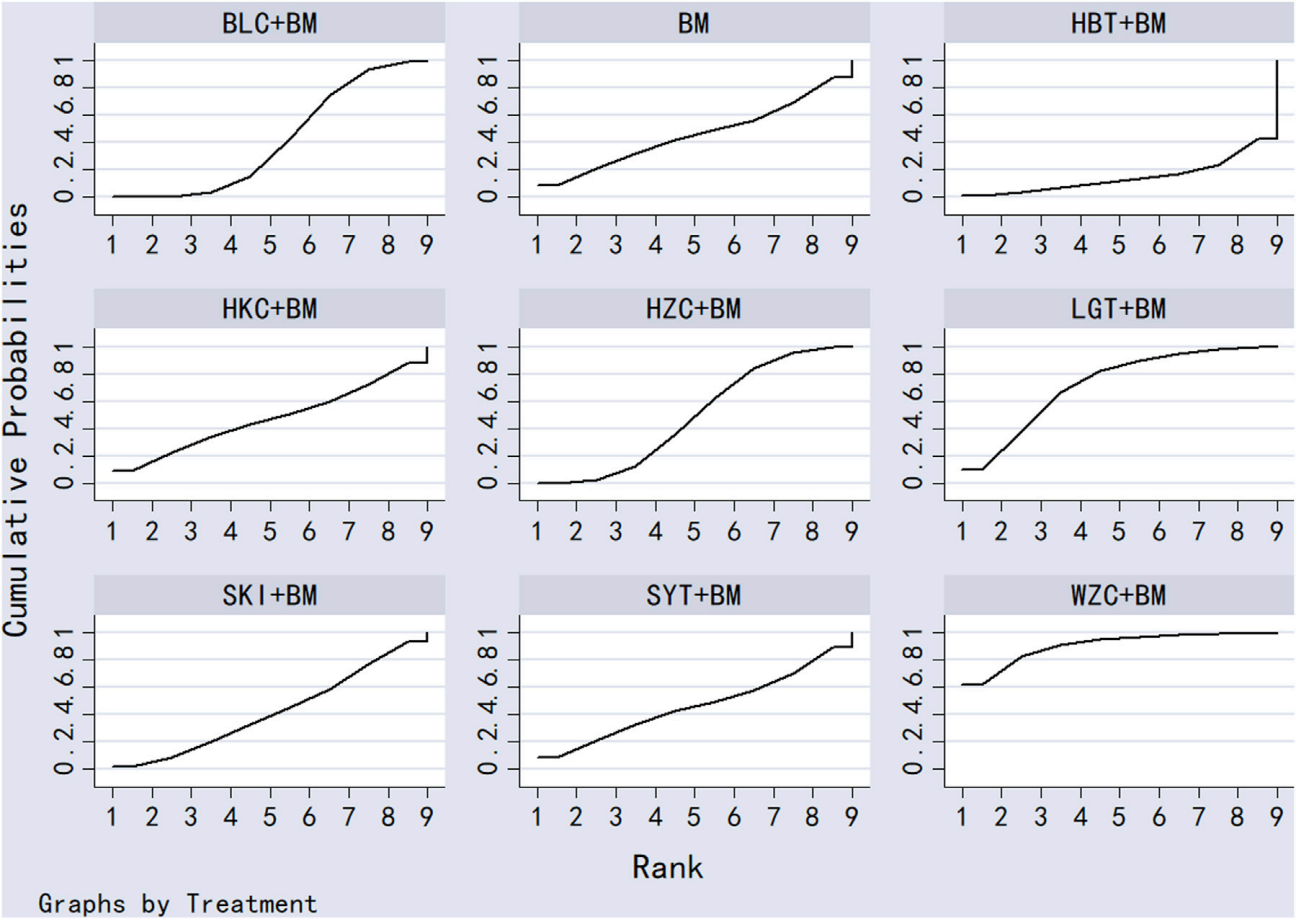
SUCRA plot of incidence of adverse reactions.

A total of 16 studies detailed adverse events across 9 treatment regimens, with no closed loops among these regimens, as depicted in the evidence network graph in Figure 13. Because all pairwise comparisons between the treatment regimens were based on indirect comparisons, inconsistency tests were unnecessary. Statistical analysis using the consistency model revealed no significant differences among the treatment regimens, as presented in Table 8. The SUCRA ranking for the incidence of adverse events was as follows: WZC + BM (90.8%) > LGT + BM (73.1%) > HZC + BM (49.1%) > HKC + BM (46.9%) > SYT + BM (46.6%) > BM (45.6%) > BLC + BM (41.5%) > SKI + BM (41.1%) > HBT + BM (15.1%). The SUCRA ranking diagram is shown in Figure 14.

**TABLE 8:** Network meta analysis results of incidence of adverse reactions.

WZC+BM	3.00 (0.28,31.94)	6.22 (0.80,48.36)	6.67 (0.23,192.62)	7.00 (0.24,205.29)	7.00 (0.24,205.85)	7.00 (0.95,51.80)	7.62 (0.60,96.99)	35.00 (0.96,1273.47)
0.33 (0.03, 3.55)	LGT+BM	2.07 (0.54, 7.90)	2.22 (0.11, 43.87)	2.33 (0.12, 46.85)	2.33 (0.12, 46.99)	2.33 (0.66, 8.23)	2.54 (0.34, 19.03)	11.67 (0.46, 298.06)
0.16 (0.02, 1.25)	0.48 (0.13, 1.84)	HZC+BM	1.07 (0.07, 16.62)	1.13 (0.07, 17.77)	1.13 (0.07, 17.83)	1.13 (0.72, 1.77)	1.23 (0.24, 6.28)	5.63 (0.28, 115.28)
0.15 (0.01, 4.33)	0.45 (0.02, 8.88)	0.93 (0.06, 14.45)	HKC+BM	1.05 (0.02, 48.67)	1.05 (0.02, 48.78)	1.05 (0.07, 15.68)	1.14 (0.05, 26.04)	5.25 (0.09, 294.61)
0.14 (0.00, 4.19)	0.43 (0.02, 8.60)	0.89 (0.06, 14.02)	0.95 (0.02,44.14)	SYT+BM	1.00 (0.02, 47.07)	1.00 (0.07, 15.21)	1.09 (0.05, 25.20)	5.00 (0.09, 284.10)
0.14 (0.00, 4.20)	0.43 (0.02, 8.63)	0.89 (0.06, 14.06)	0. 95 (0.02, 44.25)	1.00 (0.02, 47.07)	BM	1.00 (0.07, 15.26)	1.09 (0.05, 25.28)	5.00 (0.09, 284.74)
0.14 (0.02, 1.06)	0.43 (0.12, 1.51)	0.89 (0.57, 1.39)	0.95 (0.06, 14.22)	1.00 (0.07, 15.21)	1.00 (0.07, 15.26)	BLC+BM	1.09 (0.23, 5.23)	5.00 (0.25, 98.96)
0.13 (0.01, 1.67)	0.39 (0.05, 2.95)	0.82 (0.16, 4.18)	0.88 (0.04, 19.96)	0.92 (0.04, 21.29)	0.92 (0.04, 21.36)	0.92 (0.19, 4.42)	SKI+BM	4.60 (0.16, 134.10)
0.03 (0.00, 1.04)	0.09 (0.00, 2.19)	0.18 (0.01, 3.64)	0.19 (0.00, 10.69)	0.20 (0.00, 11.36)	0.20 (0.00, 11.39)	0.20 (0.01, 3.96)	0.22 (0.01, 6.35)	HBT+BM

Note: The above data are confidence intervals, and bold characters indicate statistically significant differences.

#### 3.5.7 Small sample effect estimation

**FIGURE 15:**
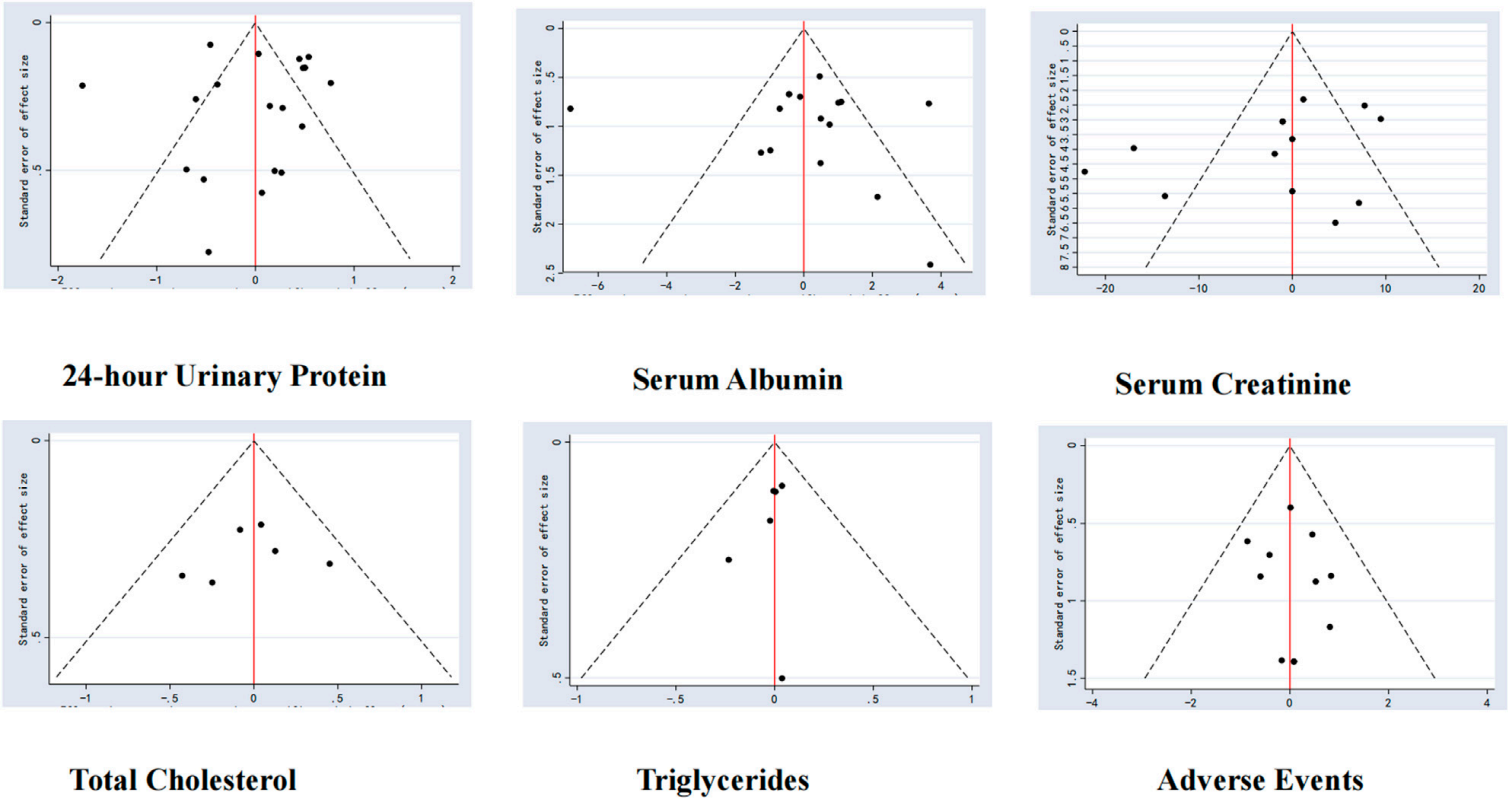
Comparison-adjusted funnel plots.

To evaluate the effects of small studies, comparison-adjusted funnel plots were created for the studies included in the analysis. The findings indicated that these funnel plots were not entirely symmetrical, suggesting the possible existence of publication bias or small-study effects within the research network (Figure 15).

## 4 Discussion

Traditional Chinese Medicine boasts a long-standing tradition in preventing and treating kidney diseases. In TCM, IMN is categorized under “edema,” “turbid urine,” and “consumptive disease” based on clinical manifestations. Numerous clinical studies have demonstrated that various Chinese herbal medicines are effective in treating IMN and are often used alongside biomedicine in clinical practice. Previous research has confirmed that combining Chinese herbal medicine with biomedicine in the treatment of IMN yields better results than using biomedicine alone (Li and Wang, 2018). However, due to the wide range of Chinese medicines used in clinical settings, identifying the most effective combination has become a focal point of current research.

Ma et al. (2023) conducted a network meta-analysis to assess the efficacy and safety of various Chinese polyherbal preparation (CCPP)s in treating idiopathic membranous nephropathy. However, their research included only a limited number of Chinese medicines, thus offering limited evidence support. This study comprehensively evaluated the effectiveness and safety of 15 types of Chinese herbal medicines in combination with biomedicine for treating IMN. These included Wuzhi capsules, Lei Gong Teng polyglycosides tablets, Shengmai injection, Shenfu Kang II capsules, Huangzhi Yishen Capsules, Maixuekang capsules, Kunxian capsules, Bailing capsules, Di Huang Ye total glycosides capsules, Qing Re Mo Shen granules, Shen Yan Kang Fu tablets, Qiqi Yi Shen capsules, Huo Ba Hua Gen tablets, Shen Kang injection, and Huang Kui capsules. To facilitate the combination of various studies for analysis, this research considered different types of biomedicines as the same intervention measure. To minimize clinical heterogeneity, studies using homemade or in-house preparations were excluded. The biomedicines included immunosuppressants (Cyclosporine A, Prednisone, Tacrolimus, Cyclophosphamide), ARBs (Candesartan, Irbesartan, Losartan Potassium, Valsartan), and ACEIs (Benazepril, Enalapril). The findings indicated that in terms of reducing 24-hour urinary protein, the most effective were Qiqi Yi Shen capsules combined with biomedicine, Leigongteng polysaccharide tablets combined with biomedicine, and Maixuekang Capsules combined with biomedicine. For reducing serum albumin, Huangzhi Yishen Capsules combined with biomedicine demonstrated the best therapeutic effect. The combination of Maixuekang Capsules and biomedicine showed the most significant reduction in triglyceride and total cholesterol levels. Meanwhile, Wuzhi capsules combined with biomedicine had the lowest incidence of adverse reactions. Studies reporting adverse events revealed that none of the Chinese herbal medicine combinations with biomedicine had a higher rate of adverse reactions compared to biomedicine alone, suggesting that these Chinese medicines do not increase the likelihood of adverse reactions. Nevertheless, the funnel plot indicated the presence of biases within this study. Several factors could account for this. Firstly, all included studies were published in Chinese and conducted in China, which might introduce regional bias. Secondly, the limited range of drugs included and the small sample sizes of some studies may have skewed the results, thus affecting the scientific rigor of our conclusions. Thirdly, the overall quality of the included studies was not ideal, potentially leading to biases. Despite the publication bias in the network meta-analysis, this study objectively and rigorously assessed the efficacy and safety of combining different Chinese medicines with biomedicines for the treatment of IMN, thereby offering clinical evidence to support this approach. The research by Ma et al. (2023) also confirmed the superior effectiveness of IMN treatment when combining Chinese and biomedicines.

Recent pharmacological studies have demonstrated that Huang Kui capsules, which are abundant in flavonoids, polysaccharides, and nucleosides, exhibit properties such as immune response inhibition, anti-inflammatory effects, and improvement of renal fibrosis (Chen et al., 2012). The primary active botanical drugss in Di Huang Ye total glycosides capsules are phenylethanoid glycosides, which are known to boost the body’s non-specific immunity. Animal studies have shown that these capsules can enhance renal function, lower inflammatory release and endothelin levels, and prevent fibrinoid necrosis and crescent formation (Liu, 2021). Leigongteng polysaccharide, derived from the roots of Tripterygium Wilford II, is known as a “herbal hormone” (Yao et al., 2010) and exhibits significant anti-inflammatory and immunoregulatory properties. It has been shown to effectively enhance renal function and reduce proteinuria (Jiang et al., 2021). Huo Ba Hua Gen tablets contain active botanical drugs such as catechins, triptolide, and tripdiolide, which possess steroid-like anti-inflammatory and immunosuppressive capabilities, effectively mitigating systemic or localized renal immune inflammatory responses and improving renal function (Zhong et al., 2021). The latest TCM clinical practice guidelines for IMN (2021) also endorse the use of Huang Kui capsules, Leigongteng polyglycosides, and Huo Ba Hua Gen tablets for treating moderate to high-risk IMN. Additionally, our subgroup analysis indicated that Leigongteng polysaccharide tablets could significantly improve 24-hour urinary protein, serum albumin, serum creatinine, and triglycerides levels in a short period. Thus, our findings suggest that Leigongteng polysaccharide tablets may be a promising Chinese medicine for the treatment of IMN.

In this study, meta-analysis demonstrated its advantages and clinical significance in the following aspects. First, it evaluated the efficacy and safety of various Chinese herbal medicines combined with Western treatments for IMN. By analyzing data from 31 RCTs, it ranked the effects of different treatment strategies on reducing 24-hour urinary protein, enhancing serum albumin, decreasing serum creatinine, improving total cholesterol, and lowering triglycerides. This comparison helped identify the most effective combined treatment regimen. Second, the study not only considered the differences in efficacy among the treatment regimens but also examined the incidence of adverse reactions. By comprehensively evaluating both efficacy and safety, the study aimed to identify a combination treatment plan that offered better results with fewer side effects. These findings suggested that traditional Chinese medicine could play a significant role as a complementary and alternative therapy to biomedicine in treating IMN, thereby providing clinicians with more reliable treatment recommendations (Zhang et al., 2024; Zihao Zhang et al., 2024).

This study had several limitations: 1) The overall quality of the included studies was suboptimal, with only 12 studies detailing their randomization methods, a single study using blinding techniques, and none being preregistered, which might introduce bias; 2) There was evidence of publication bias and small-study effects in the included studies, which could compromise the reliability of the results; 3) All the studies were conducted at single-center locations, with some having limited sample sizes, potentially affecting the external validity and accuracy of the results.

## 5 Conclusion

To summarize, this study employed network meta-analysis to establish a comprehensive network model for comparing various treatment approaches that integrated traditional Chinese medicine with biomedicine for IMN. The primary aim is to provide valuable insights for clinical decision-making in healthcare. Due to certain limitations within the included studies, it is advisable for clinical practice to choose suitable treatments based on the specific circumstances.

## Data Availability

The original contributions presented in the study are included in the article/Supplementary Material, further inquiries can be directed to the corresponding author.
