# Supplementary material for: Efficacy and safety of Chinese herbal medicines combined with biomedicine in the treatment of idiopathic membranous nephropathy: a systematic review and network meta-analysis
Source: Front Pharmacol. 2024 Oct 30;15:1391675. doi: 10.3389/fphar.2024.1391675 (PMC11557385; doi:10.3389/fphar.2024.1391675)
Supplement: Supplementary file 1 [file DataSheet1.zip › Supplementary Tables and Figures.docx]

| **Table S1.Search strategy** | |
| --- | --- |
| #1 | Glomerulonephritis, Membranous[MeSH Terms] |
| #2 | Glomerulonephritides, Membranous[Title/Abstract] |
| #3 | Membranous Glomerulonephritides[Title/Abstract] |
| #4 | Membranous Glomerulonephritis[Title/Abstract] |
| #5 | Nephropathy, Membranous[Title/Abstract] |
| #6 | Membranous Glomerulopathy[Title/Abstract] |
| #7 | Glomerulopathy, Membranous[Title/Abstract] |
| #8 | Membranous Nephropathy[Title/Abstract] |
| #9 | Extramembranous Glomerulopathy[Title/Abstract] |
| #10 | Glomerulopathy, Extramembranous[Title/Abstract] |
| #11 | Membranous Glomerulonephropathy[Title/Abstract] |
| #12 | Glomerulonephropathy, Membranous[Title/Abstract] |
| #13 | Heymann Nephritis[Title/Abstract] |
| #14 | Nephritis, Heymann[Title/Abstract] |
| #15 | Idiopathic Membranous Glomerulonephritis[Title/Abstract] |
| #16 | Glomerulonephritides, Idiopathic Membranous[Title/Abstract] |
| #17 | Glomerulonephritis, Idiopathic Membranous[Title/Abstract] |
| #18 | Idiopathic Membranous Glomerulonephritides[Title/Abstract] |
| #19 | Membranous Glomerulonephritides, Idiopathic[Title/Abstract] |
| #20 | Membranous Glomerulonephritis, Idiopathic[Title/Abstract] |
| #21 | Idiopathic Membranous Nephropathy[Title/Abstract] |
| #22 | Membranous Nephropathy, Idiopathic[Title/Abstract] |
| #23 | Nephropathy, Idiopathic Membranous[Title/Abstract] |
| #24 | OR/1-23 |
| #25 | Medicine, Chinese Traditional[MeSH Terms] |
| #26 | Traditional Chinese Medicine[Title/Abstract] |
| #27 | Chung I Hsueh[Title/Abstract] |
| #28 | Hsueh, Chung I[Title/Abstract] |
| #29 | Traditional Medicine, Chinese[Title/Abstract] |
| #30 | Zhong Yi Xue[Title/Abstract] |
| #31 | Chinese Traditional Medicine[Title/Abstract] |
| #32 | Chinese Medicine, Traditional[Title/Abstract] |
| #33 | Traditional Tongue Diagnosis[Title/Abstract] |
| #34 | Tongue Diagnoses, Traditional[Title/Abstract] |
| #35 | Tongue Diagnosis, Traditional[Title/Abstract] |
| #36 | Traditional Tongue Diagnoses[Title/Abstract] |
| #37 | Traditional Tongue Assessment[Title/Abstract] |
| #38 | Tongue Assessment, Traditional[Title/Abstract] |
| #39 | Traditional Tongue Assessments[Title/Abstract] |
| #40 | Chinese herbal medicine[Title/Abstract] |
| #41 | Chinese Herbs[Title/Abstract] |
| #42 | Chinese patent drug[Title/Abstract] |
| #43 | Oral liquid[Title/Abstract] |
| #44 | Chinese patent medicine[Title/Abstract] |
| #45 | Chinese medicine[Title/Abstract] |
| #46 | OR/23-45 |
| #47 | Randomized Controlled Trial [Publication Type] |
| #48 | Randomized Controlled Trials as Topic[MeSH Terms] |
| #49 | Clinical Trials, Randomized[Title/Abstract] |
| #50 | Trials, Randomized Clinical[Title/Abstract] |
| #51 | Controlled Clinical Trials, Randomized[Title/Abstract] |
| #52 | Randomized Controlled Trial[Title/Abstract] |
| #53 | Random*[Title/Abstract] |
| #54 | OR/47-53 |
| #55 | #24 AND #46 AND #54 |

| **Table S2.Results of a paired comparison meta-analysis of 24-hour urinary protein** | | | | | |
| --- | --- | --- | --- | --- | --- |
| Interventions | No. of studies | Heterogeneity | | WMD(95%CI) | P Value |
|  |  | I^2^ | P |  |  |
| BLC+BM vs BM | 2 | 19.6% | 0.265 | -0.48(-0.97, 0.01) | 0.055 |
| DHT+BM vs BM | 1 | NA | NA | **-0.52(-0.74, -0.30)** | **< 0.0001** |
| HBT+BM vs BM | 1 | NA | NA | **-0.57(-0.90, -0.24)** | **0.001** |
| HKC+BM vs BM | 4 | 96.30% | < 0.0001 | **-0.64(-1.24, -0.05)** | **0.035** |
| HZC+BM vs BM | 1 | NA | NA | **-0.83(-1.56, -0.10)** | **0.025** |
| KXC+BM vs BM | 1 | NA | NA | **-0.78(-0.10, -0.56)** | **< 0.0001** |
| LGT+BM vs BM | 9 | 92.50% | < 0.0001 | **-1.33(-1.92, -0.75)** | **< 0.0001** |
| MXC+BM vs BM | 1 | NA | NA | **-1.34(-2.50, -0.18)** | **0.024** |
| QQC+BM vs BM | 1 | NA | NA | **-1.77(-2.60, -0.94)** | **< 0.0001** |
| QRG+BM vs BM | 1 | NA | NA | 0.14(-0.51, 0.79) | 0.675 |
| SFC+BM vs BM | 1 | NA | NA | -0.62(-1.53, 0.29) | 0.183 |
| SKI+BM vs BM | 1 | NA | NA | **-1.2(-1.78, -0.62)** | **< 0.0001** |
| SMI+BM vs BM | 1 | NA | NA | **-0.94(-1.61, -0.27)** | **0.006** |
| SYT+BM vs BM | 2 | 51.6% | 0.151 | **-0.37(-0.57, -0.17)** | **< 0.0001** |
| WZC+BM vs BM | 2 | 0 | 0.469 | 0.30(-0.52, 1.13) | 0.473 |
| Note:NA, Data unavailable; WMD, Weighted Mean Difference; CI, Confidence interval; Bold characters indicate statistically significant differences. | | | | | |

| **Table S3. Results of a paired comparison meta-analysis of serum albumin** | | | | | |
| --- | --- | --- | --- | --- | --- |
| Interventions | No. of studies | Heterogeneity | | WMD(95%CI) | P Value |
|  |  | I^2^ | P |  |  |
| BLC+BM vs BM | 2 | 66.20% | 0.086 | **4.56(0.07, 9.04)** | **0.047** |
| DHT+BM vs BM | 1 | NA | NA | **2.85(2.67, 3.01)** | **< 0.0001** |
| HBT+BM vs BM | 1 | NA | NA | **4.11(3.27, 4.95)** | **< 0.0001** |
| HKC+BM vs BM | 3 | 0 | 0.427 | **3.43(2.76, 4.10)** | **< 0.0001** |
| HZC+BM vs BM | 1 | NA | NA | **8.23(5.78, 10.69)** | **< 0.0001** |
| KXC+BM vs BM | 1 | NA | NA | **7.62(6.71, 8.53)** | **< 0.0001** |
| LGT+BM vs BM | 9 | 91.90% | < 0.0001 | **4.91(2.90, 6.91)** | **< 0.0001** |
| MXC+BM vs BM | 1 | NA | NA | **2.94(0.41, 5.47)** | **0.023** |
| QQC+BM vs BM | 1 | NA | NA | **6.88(4.67, 9.09)** | **< 0.0001** |
| QRG+BM vs BM | 1 | NA | NA | -0.58(-3.07, 1.91) | 0.648 |
| SFC+BM vs BM | 1 | NA | NA | **4.00(2.23, 5.78)** | **< 0.0001** |
| SKI+BM vs BM | 1 | NA | NA | **6.00(2.74, 9.26)** | **< 0.0001** |
| SYT+BM vs BM | 2 | 35.70% | 0.212 | **4.95(3.43, 6.47)** | **< 0.0001** |
| WZC+BM vs BM | 1 | NA | NA | 2.00(-1.33, 5.33) | 0.239 |
| Note:NA, Data unavailable; WMD, Weighted Mean Difference; CI, Confidence interval; Bold characters indicate statistically significant differences. | | | | | |

| **Table S4. Results of a paired comparison meta-analysis of serum creatinine** | | | | | |
| --- | --- | --- | --- | --- | --- |
| Interventions | No. of studies | Heterogeneity | | WMD(95%CI) | P Value |
|  |  | I^2^ | P |  |  |
| BLC+BM vs BM | 1 | NA | NA | **-12(-22.43, -1.57)** | **0.024** |
| HBT+BM vs BM | 1 | NA | NA | **-8.90(-13.12, -4.68)** | **< 0.0001** |
| HKC+BM vs BM | 3 | 0 | 0.782 | 1.84(-1.26, 4.93) | 0.244 |
| HZC+BM vs BM | 1 | NA | NA | -2.17(-17.87, 13.53) | 0.786 |
| KXC+BM vs BM | 1 | NA | NA | **-9.98(-15.24, -4.73)** | **< 0.0001** |
| LGT+BM vs BM | 6 | 92.6 | < 0.0001 | **-12.38(-23.80, -0.96)** | **0.034** |
| QQC+BM vs BM | 1 | NA | NA | **-5.97(-9.55, -2.39)** | **0.001** |
| QRG+BM vs BM | 1 | NA | NA | -6.42(-13.99, 1.15) | 0.096 |
| SKI+BM vs BM | 1 | NA | NA | -2.00(-7.99, 3.99) | 0.513 |
| SYT+BM vs BM | 2 | 0 | 0.399 | -0.11(-6.97, 6.74) | 0.974 |
| WZC+BM vs BM | 2 | 0 | 1.000 | -1.00(-6.94, 4.94) | 0.741 |
| Note:NA, Data unavailable; WMD, Weighted Mean Difference; CI, Confidence interval; Bold characters indicate statistically significant differences. | | | | | |

| **Table S5. Results of a paired comparison meta-analysis of total cholesterol** | | | | | |
| --- | --- | --- | --- | --- | --- |
| Interventions | No. of studies | Heterogeneity | | WMD(95%CI) | P Value |
|  |  | I^2^ | P |  |  |
| HBT+BM vs BM | 1 | NA | NA | **-0.88(-1.01, -0.75)** | **< 0.0001** |
| HKC+BM vs BM | 2 | 0 | 0.56 | -0.08(-0.42, 0.27) | 0.661 |
| HZC+BM vs BM | 1 | NA | NA | -0.53(-1.44, 0.38) | 0.255 |
| LGT+BM vs BM | 4 | 27.9% | 0.244 | **-0.34(-0.62, -0.06)** | **0.017** |
| MXC+BM vs BM | 1 | NA | NA | **-1.20(-1.96, -0.44)** | **0.002** |
| QRG+BM vs BM | 1 | NA | NA | **-0.81(-1.59, -0.03)** | **0.041** |
| SFC+BM vs BM | 1 | NA | NA | -0.59(-1.76, 0.58) | 0.323 |
| Note:NA, Data unavailable; WMD, Weighted Mean Difference; CI, Confidence interval; Bold characters indicate statistically significant differences. | | | | | |

| **Table S6. Results of a paired comparison meta-analysis of triglycerides** | | | | | |
| --- | --- | --- | --- | --- | --- |
| Interventions | No. of studies | Heterogeneity | | WMD(95%CI) | P Value |
|  |  | I^2^ | P |  |  |
| HBT+BM vs BM | 1 | NA | NA | -3.00(-0.68, 0.08) | 0.122 |
| HKC+BM vs BM | 2 | 0 | 0.946 | -0.09(-0.23, 0.06) | 0.246 |
| LGT+BM vs BM | 4 | 0 | 0.786 | **-0.54(-0.69, -0.39)** | **< 0.0001** |
| MXC+BM vs BM | 1 | NA | NA | **-0.80(-1.12, -0.48)** | **< 0.0001** |
| QRG+BM vs BM | 1 | NA | NA | -0.02(-0.71, 0.67) | 0.955 |
| SFC+BM vs BM | 1 | NA | NA | -0.24(-0.86, 0.38) | 0.448 |
| Note:NA, Data unavailable; WMD, Weighted Mean Difference; CI, Confidence interval; Bold characters indicate statistically significant differences. | | | | | |

| **Table S7. Results of a paired comparison meta-analysis of adverse events incidence** | | | | | |
| --- | --- | --- | --- | --- | --- |
| Interventions | No. of studies | Heterogeneity | | RR(95%CI) | P Value |
|  |  | I^2^ | P |  |  |
| BLC+BM vs BM | 1 | NA | NA | 0.85(0.56, 1.28) | 0.426 |
| HBT+BM vs BM | 1 | NA | NA | 1.00(0.70, 1.43) | 1.000 |
| HKC+BM vs BM | 1 | NA | NA | 1.00(0.69, 1.44) | 1.000 |
| HZC+BM vs BM | 1 | NA | NA | 0.93(0.67, 1.30) | 0.688 |
| LGT+BM vs BM | 8 | 0 | 1.000 | 0.99(0.90, 1.10) | 0.899 |
| SKI+BM vs BM | 1 | NA | NA | 1.00(0.65, 1.53) | 0.996 |
| SYT+BM vs BM | 1 | NA | NA | 1.04(0.71, 1.54) | 0.837 |
| WZC+BM vs BM | 3 | 0 | 1.000 | 1.00(0.81, 1.24) | 0.989 |
| Note:NA, Data unavailable; RR, Relative risk; CI, Confidence interval;Bold characters indicate statistically significant differences. | | | | | |

| **Table S8. Results of subgroup analysis of Leigongteng polysaccharide tablets** | | | | | | |
| --- | --- | --- | --- | --- | --- | --- |
| Outcomes | Course | No. of studies | Heterogeneity | | WMD/RR(95%CI) | P Value |
|  |  |  | I^2^ | P |  |  |
| 24-hour urinary protein | 4 months | 1 | NA | NA | **-1.65(-2.06, -1.24)** | **< 0.0001** |
|  | 6 months | 4 | 96.40% | < 0.0001 | **-1.75(-2.94, -0.55)** | **0.004** |
|  | 12 months | 4 | 0 | 0.452 | **-0.71(-1.00, -0.43)** | **< 0.0001** |
| Serum albumin | 4 months | 1 | NA | NA | **8.46( 6.95, 9.97)** | **< 0.0001** |
|  | 6 months | 4 | 95.50% | < 0.0001 | **3.62( 0.13, 7.10)** | **0.042** |
|  | 12 months | 4 | 0 | 0.452 | **4.96( 3.92, 6.00)** | **< 0.0001** |
| Serum creatinine | 4 months | 1 | NA | NA | **-24.82(-32.58, -17.07)** | **< 0.0001** |
|  | 6 months | 3 | 94.60% | < 0.0001 | -16.38(-37.65, 4.89) | 0.131 |
|  | 12 months | 2 | 0 | 0.926 | -0.23( -4.76, 4.30) | 0.92 |
| Total cholesterol | 6 months | 1 | NA | NA | -0.59(-1.30, 0.12) | 0.101 |
|  | 12 months | 3 | 44.40% | 0.165 | -0.30( -0.60, 0.01) | 0.06 |
| Triglycerides | 6 months | 1 | NA | NA | **-0.56(-0.89, -0.24)** | **0.001** |
|  | 12 months | 3 | 0 | 0.595 | **-0.53( -0.70, -0.37)** | **< 0.0001** |
| Adverse events incidence | 4 months | 1 | NA | NA | 0.98(0.75, 1.27) | 0.881 |
|  | 6 months | 4 | 0 | 0.996 | 1.01( 0.88, 1.15) | 0.944 |
|  | 12 months | 3 | 0 | 0.846 | 0.98(0.82, 1.17) | 0.813 |
| Note:NA, Data unavailable; WMD, Weighted Mean Difference; RR, Relative risk; CI, Confidence interval; Bold characters indicate statistically significant differences. | | | | | | |

| **Table S9. Detailed Composition of Each Studied Preparation and Corresponding Reporting in Original Studies** | | | | | | | | | | | |
| --- | --- | --- | --- | --- | --- | --- | --- | --- | --- | --- | --- |
| Medicine Name | Related Ingredients | Full Species Name (Including Authority and Family) | Part Used (Aerial Parts, Roots, Bark, etc.), Fresh, Dried, Fermented, etc. | Dosage Form (Tablet, Capsule, etc.) | Usage and Dosage | Place of Origin | Harvesting and Processing | Chemical Components | Manufacturer Name and Supplier | Approval Number | Implementation Standard |
| Wuzhi Capsules | Schisandra Chinensis | Schisandra Chinensis (Turcz.) Baill. [Magnoliaceae; Schisandrae Chinensis Fructus]. | the desiccative ripe fruit | Capsule, each containing 11.25mg of Schisandrin A | Oral. 2 capsules per dose, 3 times daily. | Mainly produced in Liaoning, Heilongjiang, Jilin provinces. | Harvest mature fruits in autumn, dry in the sun or steam and then dry, remove stems and impurities. | Mainly contains lignans, volatile oil, polysaccharides, organic acids, terpenoids, flavonoids, etc. | Sichuan Hezheng Pharmaceutical Co., Ltd. | National Medicine Standard Z10983013 | National Drug Supervision Bureau WS3-B-2094-96-2002 |
| Leigongteng polysaccharide tablet | Tripterygium wilfordii | Tripterygium wilfordii Hook.f. [Magnoliaceae; Schisandrae Chinensis Fructus]. | Roots | Tablet | Oral. 1-1.5 mg per kg body weight daily, divided into 3 doses after meals, or as prescribed. | Found in shady, moist, and fertile mountain slopes, valleys, streamside shrubs, and secondary mixed forests. Distributed in Guangdong, Fujian, Taiwan, Zhejiang, etc. | Harvest in summer and autumn, dig up roots, clean off soil, dry in the sun, or peel and dry, cut into thick slices, use raw. | Contains alkaloids such as triptolide, tripterine, celastrol, and more, as well as diterpenoids, triterpenoids, and other compounds. | Zhejiang Pulokangyu Natural Pharmaceutical Co., Ltd. | National Medicine Standard Z33020778 | Ministry of Health Traditional Chinese Medicine Formulation Seventeenth Volume WS3-B-3350-98 |
| Shengmai Injection | Panax ginseng | Panax ginseng C.A.Mey.[Araliaceae; Talinum paniculatum (Jacq.) Gaertn.]. | Cultivated ginseng steamed and dried roots and rhizomes. | Injection | Intravenous infusion. 25-60 ml per dose, diluted with 250-500 ml of 5% glucose injection, or as prescribed. | Produced in Heilongjiang, Jilin, Liaoning. Notable regions include Fusong, Ji'an, Changbai, Jingyu in Jilin, Huanren, Kuandian, Xinbin, Qingyuan in Liaoning, and Yilan in Heilongjiang. | Harvest in autumn, clean, steam, and dry. | Contains saponins and volatile oils, fatty acids, amino acids, sterols, flavonoids, etc. | Sichuan Yibin Wuliangye Group Yibin Pharmaceutical Co., Ltd. | National Medicine Standard Z51022475 | State Food and Drug Administration National Drug Standard WS3-B-2865-98-201 |
|  | Ophiopogon japonicus | Ophiopogon japonicus (Thunb.) ker-Gawl. [Asparagaceae; Ophiopogon japonicus (L. f.) Ker Gawl.]. | Tuberous roots |  |  | Grows in shady, moist areas below 2000 meters altitude, under forests or beside streams. Distributed in Shandong, Anhui, Zhejiang, etc. | Harvest in the third year of cultivation in early summer. Dig up the roots, clean off soil, dry for 3-4 days, store in a ventilated place to regain moisture, steam, and dry. | Contains various steroidal saponins, primarily ophiopogonin A and ophiopogonin B, along with isoflavonoids and other compounds. |  |  |  |
|  | Schisandra Chinensis | Schisandra Chinensis (Turcz.) Baill. [Magnoliaceae; Schisandrae Chinensis Fructus]. | Dried mature fruit |  |  | Mainly produced in Liaoning, Heilongjiang, Jilin provinces. | Harvest mature fruits in autumn, dry in the sun or steam and then dry, remove stems and impurities. | Mainly contains lignans, volatile oil, polysaccharides, organic acids, terpenoids, flavonoids, etc. |  |  |  |
| Shenfukang II capsule | Smilax glabra | Smilax glabra Roxb. [Smilacaceae; Smilax glabra Roxb]. | Rootstock | Capsule | Oral. 4-6 capsules per dose, 3 times daily. | Grows in mountains, slopes, valleys, sparse forests, and shrublands or riverbank forest edges. Distributed in Guangdong, Hainan, Guangxi, Fujian, etc. | Harvest in summer and autumn, remove fibrous roots, wash, and dry; or cut into thin slices while fresh and dry. | Contains compounds such as smilaxin, isoflavones, carotenoids, succinic acid, palmitic acid, isosmilacin, smilaxin, sterol-3-O-B-D-glucopyranoside, quercetin, isoquercetin, tannins, resins, diosgenin and trace volatile oil. | Jilin Aodong Group Liyuan Pharmaceutical Co., Ltd. | National Medicine Standard Z22021224 | Chinese Pharmacopoeia 2020 Edition Part 1 |
|  | Sophora japonica | Sophora japonicaL. Fabaceae; Robinia pseudoacacia L.]. | Flower |  |  | Widely cultivated in northern and southern China, especially in the Loess Plateau and North China Plain. | Harvest flowers or flower buds in summer when they bloom, dry promptly, remove branches, stems, and impurities. | Contains rutin (10%-28%), triterpene saponins, glucose, glucuronic acid, rhamnose, iso-rhamnosyl-3-rutinoside, kaempferol-3-rutinoside, quercetin, saponins, Sophora japonica saponins, Sophora japonica diol, etc. |  |  |  |
|  | Imperata cylindrica | Imperata cylindrica Beauv.var. major（ Nees）C.E.Hubb [Poaceae;Imperata koenigii (Retz.) P. Beauv.]. | [Dried rhizome](https://baike.baidu.com/item/%E6%A0%B9%E8%8C%8E/5537329?fromModule=lemma_inlink) |  |  | Grows on slopes and wastelands. Distributed throughout the country. | Harvest in spring and autumn, clean, sun-dry, remove fibrous roots and membranous leaf sheaths, bundle into small packages. | Contains triterpenoids such as cylindrin, imperanene, fernsone, isofernenol, simirol, and soluble calcium, with sugars at 18.8%, mainly glucose, sucrose, small amounts of fructose, and xylose. Also contains monoacids and their potassium salts, carotenoids, chlorophyll, and pulsatilla saponin, palmitic acid, etc. |  |  |  |
|  | Leonurus japonicus | Leonurus japonicus Houtt. [Lamiaceae; Leonurus japonicusHoutt]. | Aerial parts of the plant |  |  | Grows on slopes, grasslands, roadsides, etc. Distributed throughout the country. | Harvest stems and leaves in summer when they are flourishing, before or just after flowering, cut into sections, and dry in the sun. | Whole plant contains leonurine, stachydrine, leonuridine, and leonuride diterpenoids. |  |  |  |
|  | Agastache rugosa | Agastache rugosa (Fisch. & C. A. Mey.) Kuntze [Lamiaceae; Agastache rugosa (Fisch. et Mey.) O. Ktze.]. | Whole plant |  |  | Grows on slopes or roadsides. Distributed in Heilongjiang, Jilin, Liaoning, Hebei, etc. | Harvest the first time in June to July when blooming, and the second time in October. Remove impurities, cut into sections, and dry in the sun or shade. | Contains methyl chavicol, anethole, anisaldehyde, limonene, para-methoxy cinnamaldehyde, etc. |  |  |  |
| Huangzhi Yishen capsules | Astragalus membranaceus | Astragalus membranaceus Bge. var. mongholicus (Bge.) Hsiao [Leguminosae; Astragali Radix]. | Root | Capsule | Oral. 5 capsules per dose, 3 times daily. | Grows on slopes or roadsides. Distributed in Heilongjiang, Jilin, Liaoning, Hebei, etc. | Harvest the first time in June to July when blooming, and the second time in October. Remove impurities, cut into sections, and dry in the sun or shade. | Contains methyl chavicol, anethole, anisaldehyde, limonene, para-methoxy cinnamaldehyde, etc. | Leiyunshang Pharmaceutical Group Co., Ltd. | National Medicine Standard Z20020086 | State Food and Drug Administration National Drug Standard WS-755 (Z-41)-2005 (Z) |
|  | Lycium barbarum L. | Lycium barbarum L. [Solanaceae; Lycii Fructus.]. | Mature fruit |  |  | Distributed in Heilongjiang, Jilin, Liaoning, Hebei, Inner Mongolia, etc. | Wild Astragalus can be harvested in spring and autumn, remove soil and fibrous roots, cut off the root head, sun-dry until 70-80% dry, and grade according to thickness and length. Cultivated Astragalus should be harvested after 3 years. | Contains astragaloside, astragaloside, calycosin glucoside, calycosin, isoastragaloside I, isoastragaloside II, triterpenoid saponins, flavonoids, and polysaccharides. |  |  |  |
|  | Dioscorea opposita Thunb. | Dioscorea opposita Thunb. [Dioscoreaceae; Dioscoreae Rhizoma.]. | Rootstock |  |  | Distributed in Hebei, Inner Mongolia, Shaanxi, etc. | [Harvest in summer and autumn when the fruit turns orange-red, air-dry until the skin wrinkles, then sun-dry until the outer skin is hard and the pulp is soft, remove the stems; or dry at low temperature with hot air, remove the stems.](http://www.zhongyoo.com/name/liuhuang_1443.html) | Contains polysaccharides, fatty acids, betaine, carotene, riboflavin, niacin, amino acids, vitamin C, and various trace elements. Also contains atropine, beta-carotene, cryptoxanthin, zeaxanthin, scopoletin, etc. |  |  |  |
|  | Coix lacryma-jobi L. var. mayuen | Coix lacryma-jobi L. var. mayuen (Roman.) Stapf [Gramineae; Coicis Semen.]. | Dried mature seeds |  |  | Grows on sunny slopes or forest edges, often cultivated. Mainly produced in Henan, Shaanxi, etc. | Harvest after stems and leaves wither in winter, remove the root head, wash, peel and remove fibrous roots, sulfur fumigation and drying, or select plump and straight roots, soak in water until fully moist, sulfur fumigation, cut both ends, roll into cylindrical shape with wooden board, sun-dry, polish, commonly called "glossy yam". | [Contains dopamine, dioscorin, protodioscin II, sitosterol, cholesterol, diosgenin, ergosterol, quinine acid, brassicasterol, cholesterol, yam saponin, allantoin, brassicasterol, hexacosanoic acid, beta-sitosterol; also contains free amino acids, starch, tannins, mucilage, glycoproteins, polyphenol oxidase, etc.](http://www.zhongyoo.com/name/zonglv_1041.html) |  |  |  |
|  | Scrophularia ningpoensis Hemsl. | Scrophularia ningpoensis Hemsl. / Scrophularia buergeriana Miq. (S. oldhami Oliv.) [Scrophulariaceae; Scrophulariae Radix]. | Dried root |  |  | Grows by rivers, streams, or in shady, moist valleys, prefers warm, humid areas. Widely produced in Fujian, Jiangsu, etc. | Harvest the plants in autumn when the fruits are mature, sun-dry, thresh to collect seeds, sun-dry again, remove the husks, yellow-brown seed coats, and impurities, and collect the seeds. | Contains coixenolide, palmitic acid, linoleic acid, coix polysaccharide A, coix polysaccharide B, coix polysaccharide C, etc. |  |  |  |
|  | Glehnia littoralis | Glehnia littoralis Fr. Schmidt ex Miq. [Umbelliferae; Glehniae Radix]. | Dried root |  |  | Prefers moist soil. Distributed in Heilongjiang, Jilin, Liaoning, Hebei, Inner Mongolia, etc. | Dig up roots in October-November, remove stems, leaves, and soil, peel off the buds for seed cultivation, sun-dry the roots until half dry and the inside turns black, cut off the root head and fibrous roots, sweat for 3-4 days, then sun-dry or dry. | Contains harpagoside (70%-80%), 8-methylp-coumaroyl, harpagoside, all are blackening substances. Also contains harpagide, scroviscoside, scrophuloside, pinoresinol diglucoside, scrophularin, geniposidic acid, sesamin F, caffeoyl veronicoside, verbascoside, etc. |  |  |  |
|  | Eclipta prostrata L. | Eclipta prostrata L. [Compositae; Ecliptae Herba.]. | Dried aerial parts of the plant |  |  | Cultivated in fertile, loose sandy soil or wild on coastal sandy beaches. Distributed in Liaoning, Hebei, Shandong, etc. | Harvest in summer and autumn, remove fibrous roots, wash, air-dry, scald in boiling water, remove outer skin, dry. Or wash and dry directly. | Contains furanocoumarins, including psoralen, isoimperatorin, umbelliferone, scopoletin, bergapten, imperatorin, and polysaccharides. Reportedly also contains volatile oils and amino acids. |  |  |  |
|  | Eucommia ulmoides Oliv. | Eucommia ulmoides Oliv. [Eucommiaceae; Eucommiae Cortex.]. | Bark |  |  | Grows along roadsides, in wetlands, along ditches or in fields. Distributed throughout most parts of China. | Harvest when in bloom, dry. | [Contains 1.32% saponins, about 0.08% nicotine, tannins, vitamin A, wedelolactone, various thiophene compounds, leaves contain 2-(butyn-2-yl)-5-(vinyl-2-ynyl) thiophene, ecliptalactone, desmethylecliptalactone, desmethylecliptalactone-7-glucoside, aerial parts petroleum ether extract contains sitosterol, plant sterol A, and beta-amyrin; ethanol extract contains luteolin-7-O-glucoside, plant sterol A glucoside and a triterpene acid glucoside.](http://www.zhongyoo.com/name/dingxiang_39.html) |  |  |  |
|  | Panax notoginseng | Panax notoginseng (Burk.) F.H. Chen [Araliaceae; Panax notoginseng (Burkill) F. H. Chen ex C. Y. Wu & K. M. Feng]. | Root and rhizome |  |  | Grows in mountain forests or cultivated. Distributed in Sichuan, Shaanxi, Gansu, etc. | Peel off bark from April to June, scrape off rough outer skin, stack for sweating until inner bark turns purple-brown, dry. | [Contains pinoresinol diglucoside, syringin, aucubin, chlorogenic acid, geniposidic acid, and eugenol. Recent studies report branch bark contains nonacosane, octacosanol, beta-sitosterol, betulin, betulinic acid, and ursolic acid.](http://www.zhongyoo.com/name/dingxiang_39.html) |  |  |  |
|  | Leonurus japonicus Houtt. | Leonurus japonicus Houtt. [Lamiaceae; Leonurus japonicus Houtt.]. | Whole plant |  |  | Wild under forest cover on mountain slopes. Now widely cultivated on slopes or gentle hills at altitudes of 800-1000 meters, preferring loose, humus-rich acidic soil. Distributed in Guangxi, Sichuan, Yunnan, etc. | Harvest 4 years after planting, dig in autumn before flowering for "Spring Seven", full and high quality; dig in November after seeds mature for "Winter Seven", looser and lower quality. Remove aerial stems and soil, cut off root head and fibrous roots, wash, dry. | Mainly contains ginsenosides Rg₁, quercetin, acetic acid, elemene, ginsenoside Re, leucine, beta-sitosterol-D-glucoside, ginsenoside, notoginsenosides and 12 monomer saponins including hemostatic notoginin. Also contains volatile oils and various trace elements. |  |  |  |
|  | Hirudo niponica Whitman | Hirudo niponica Whitman [Hirudinidae; Hirudo]. | Dried body |  |  | Grows on slopes, grasslands, roadsides, etc. Distributed throughout China. | Harvest in summer when stems and leaves are flourishing, before or just after flowering, cut into sections, dry in the sun. | Whole plant contains leonurine, stachydrine, leonuridine, and leonuride diterpenoids. |  |  |  |
|  | Cryptotympana pustulata Fabricius | Cryptotympana pustulata Fabricius [Cicadidae; Cicadae Periostracum.]. | Exuviae (shed skin) |  |  | Inhabit ditches and rice paddies, sucking blood from humans and animals. Widely distributed in southern and northern China. | Catch in summer and autumn, wash, kill with lime or white wine, or scald to death with boiling water, dry in the sun or at low temperature. | Mainly contains proteins. Also contains various trace elements such as iron, manganese, zinc. Fresh leech saliva contains an anticoagulant substance called hirudin. |  |  |  |
|  | Plantago asiatica L. | Plantago asiatica L. [Plantaginaceae; Plantaginis Herba.]. | Whole plant |  |  | Adults usually live on broadleaf trees such as poplar, willow, maple, and apple, pear, peach trees. Nymphs hatch and fall to the ground from tree branches, undergoing several molts before emerging as adults. Distributed in areas south of Liaoning. | Collect from tree trunks or ground in summer and autumn, remove soil, and dry. | [Mainly contains chitin, 24 kinds of amino acids, and a large amount of keratin and chitosan.](http://www.zhongyoo.com/name/dingxiang_39.html) |  |  |  |
|  | Achyranthes bidentata Bl. | [Achyranthes bidentata Bl. [Amaranthaceae; Achyranthes bidentata Blume].](https://tian.nju.edu.cn/plantae/10/families/xianke.htm) | Root |  |  | Grows on slopes, roadsides, field ridges, and among grass by streams. Distributed in most provinces of China. | Harvest in summer, remove soil, and dry or use fresh. | [Contains plantain glycosides, high plantain glycosides, aucubin, ursolic acid, beta-sitosterol, stigmasterol, plantain flavonoid glycosides, eugenol glycosides, plantain glycosides, large plantain glycosides, etc.](http://www.zhongyoo.com/name/hupo_1506.html) |  |  |  |
| Maixuekang capsules | Hirudo niponica Whitman | Hirudo niponica Whitman [Hirudinidae; Hirudo]. | Dried body | Capsule | Oral. 2-4 capsules per dose, 3 times daily. | Inhabit ditches and rice paddies, sucking blood from humans and animals. Widely distributed in southern and northern China. | Catch in summer and autumn, wash, kill with lime or white wine, or scald to death with boiling water, dry in the sun or at low temperature. | Mainly contains proteins. Also contains various trace elements such as iron, manganese, zinc. Fresh leech saliva contains an anticoagulant substance called hirudin. | Chongqing Dupont Pharmaceutical Co., Ltd. | National Medicine Standard Z10970056 | New Drug Approval Standard Volume 42 WS3-97(X-87)-2002Z |
| Kunxian capsules | Tripterygium hypoglaucum | Tripterygium hypoglaucum (Levl.) Hutch [Celastraceae; RADIX TRIPTERYGII HYPOGLAUCI]. | Root | Capsule | Oral. 2 capsules per dose, 3 times daily after meals. One course of treatment is one month, can be continuously taken for 3 courses. | Found in sunny shrubs or under sparse forests. Distributed in Zhejiang, Jiangxi, Hunan, Sichuan, Guizhou, Yunnan. | Harvest in autumn, wash, slice, and sun-dry. | Contains diterpenes, triterpenes, and sesquiterpene alkaloids, as well as flavonoids, steroids, tannins, sugars, etc. | Guangzhou Baiyunshan Chenliji Pharmaceutical Co., Ltd. | National Medicine Standard Z20060267 | National Food and Drug Administration National Drug Standard YBZ07522006 |
|  |  |  |  |  |  |  |  |  |  |  |  |
|  |  |  |  |  |  |  |  |  |  |  |  |
|  | Epimedium Brevicornu Max-Im | Epimedium Brevicornu Max-Im [Hirudinidae; Hirudo]. | Leaves |  |  | Grows in bamboo forests or crevices in rocks by mountain roads. Distributed in Shaanxi, Yunnan, Guizhou, Sichuan, etc. | Harvest leaves in spring and summer, wash, and dry in the sun. | Contains icariin, epimedin A, epimedin B, epimedin C, etc. |  |  |  |
|  | Lycium barbarum L. | Lycium barbarum L. [Solanaceae; Lycii Fructus.]. | Mature fruit |  |  | Distributed in Hebei, Inner Mongolia, Shaanxi, etc. | Harvest in summer and autumn when the fruit turns orange-red, air-dry until the skin wrinkles, then sun-dry until the outer skin is hard and the pulp is soft, remove the stems; or dry at low temperature with hot air, remove the stems. | Contains polysaccharides, fatty acids, betaine, carotene, riboflavin, niacin, amino acids, vitamin C, and various trace elements. Also contains atropine, beta-carotene, cryptoxanthin, zeaxanthin, scopoletin, etc. |  |  |  |
|  | Cuscuta chinensis Lam. | Cuscuta chinensis Lam. [Cuscuta; Cuscuta chinensis Lam.]. | Mature seeds |  |  | Grows on slopes, roadsides, field edges, wastelands, and among shrubs, often parasitic on legumes, composites, and chenopods, especially common on soybeans. Distributed in most parts of China. Main production areas are Liaoning, Heilongjiang, Jilin, Inner Mongolia, Shandong, Hebei, Shanxi, etc. | Harvest in mid-October when the fruit shells turn yellow and about 1/3 of the soybean plants have withered, cut down the host plants, sun-dry, thresh, and separate the seeds. Clean and dry the seeds. For wine-prepared Cuscuta seeds, cook the seeds in water until they crack, stir continuously, absorb the water, add yellow wine and flour, mix well, press into large sheets, cut into squares, and dry (for every 100 kg of Cuscuta seeds, use 15 kg of yellow wine and 15 kg of flour). | Contains quercetin, astragalin, hyperoside, kaempferol-3-O-beta-D-galactoside-7-O-beta-glucoside, kaempferol-3-O-beta-D-glucopyranoside, kaempferol, new cuscutin, cuscuta polysaccharides, etc. Also contains cuscuta amine, cuscutin A, cuscutin B, arbutin, chlorogenic acid, caffeic acid, p-coumaric acid. Additionally contains potassium, calcium, phosphorus, sulfur, iron, copper, manganese, selenium, molybdenum, and other trace elements, as well as essential amino acids valine, methionine, isoleucine, etc. |  |  |  |
| Bailing capsules | Cordyceps sinensis | Cordyceps sinensis (Berk.) Sacc. [Clavicipitaceae; Cordyceps]. | Fruiting body and larval body of the fungus | Capsule | Oral. 5-15 capsules per dose, 3 times daily. For chronic renal insufficiency: 10 capsules per dose, 3 times daily; treatment course of 8 weeks. | Found in high-altitude mountainous areas, grasslands, river valleys, and grass clusters. Distributed in Qinghai, Sichuan, Gansu, etc. | Harvest in early summer when fruiting bodies emerge from the soil, dry to 60-70% dry, remove fibrous attachments and impurities, sun-dry or dry at low temperature. | Contains cholesterol, adenosine, ergosterol, crude protein, amino acids, fats, D-mannitol, cordycepin, galactomannan, cordycepic acid, ergosterol, cordyceps polysaccharides, alkaloids, uracil, adenine, adenosine, vitamin B12, and various trace elements. Cordycepic acid and cordycepin are the main active substances. | Hangzhou Zhongmei Huadong Pharmaceutical Co., Ltd. | National Medicine Standard Z10910036 | Chinese Pharmacopoeia 2020 Edition Part 1 |
| Dihuang Ye Total Glycoside capsules | Rehmannia glutinosa | Rehmannia glutinosa (Gaetn.) Libosch. [Scrophulariaceae; Rehmannia glutinosa (Gaertn.) Libosch. ex Fisch. & C. A. Mey.]. | Leaves | Capsule | Oral. 2 capsules per dose, 2 times daily. Treatment course of 8 weeks. | Distributed in Hebei, Shanxi, Inner Mongolia, Liaoning, Jiangsu, Zhejiang, Anhui, Shandong, Henan, Hubei, Hunan, Sichuan, Shaanxi, etc. | Mainly using multifunctional extraction tanks for reflux extraction. | Contains iridoids, violetone compounds, and phenylethanoid compounds. | Sichuan Mediking Pharmaceuticals Co., Ltd. | National Medicine Standard Z20070051 | National Food and Drug Administration National Drug Standard YBZ02022007-2010Z |
| Qing Re Mo Shen granules | Atractylodes lancea | Atractylodes lancea (Thunb.) DC. [Asteraceae; Atractylodis Rhizoma]. | Rhizome | Granules | Dissolve in hot water. 12g per dose, 3 times daily. | Found in hilly thickets and grass clusters. Distributed in Henan, Shandong, etc. | Harvest in spring and autumn, remove soil, sun-dry, and remove fibrous roots. | Contains atractylol, camphene, atractylone, patchoulene, elemene, atractylol acetate, and dehydrocostus lactone. Also contains volatile oil, mainly consisting of atractylon, atractylone, and atractylolactone. | Shanghai Longhua Hospital | Shanghai Medicine Standard Z04170943 |  |
|  | Atractylodes macrocephala | Atractylodes macrocephala Koidz [Asteraceae; Atractylodis Macrocephalae Rhizoma]. | Rhizome |  |  | Grows on slopes, forest edges, and in shrub forests. Distributed in Anhui, Zhejiang, Jiangxi, etc. | Harvest after frost and before winter solstice, remove stems and soil, dry or sun-dry, and remove fibrous roots. Dried ones are called "dried atractylodes"; sun-dried ones are called "raw sun-dried atractylodes", also known as "winter atractylodes". | Contains atractylon, mannan AM-3, alpha-humulene, beta-selinene, borneol, double atractylone, scopoletin, gamma-sitosterol, etc. |  |  |  |
|  | Codonopsis pilosula | Codonopsis pilosula (Franch.) Nannf. [Campanulaceae; Codonopsis Radix]. | Root |  |  | Grows at altitudes of 900-2300 meters in forest edge shrubs, now widely cultivated. Distributed in Hubei, Hunan, Sichuan, Guizhou, etc. | Harvest roots older than 3 years in autumn, wash, and sun-dry. | Contains volatile oil, baicalin glucoside, trace alkaloids, amino acids, polysaccharides, and saponins, as well as eugenol, n-hexyl-beta-D-glucopyranoside, taraxerol, and lupeol. |  |  |  |
|  | Salvia miltiorrhiza | Salvia miltiorrhiza Bunge [Lamiaceae; Salviae miltiorrhizae radix et rhizoma] | Root and rhizome |  |  | Grows on grassy slopes, under forests, and by streams. Distributed in Gansu, Sichuan, Guizhou, etc. | Harvest roots in autumn, remove stems, leaves, fibrous roots, and soil, sun-dry. | Contains tanshinone I, tanshinone IIA, tanshinone IIB, cryptotanshinone, shikonic acid B, cryptotanshinone, dihydrotanshinone, tanshinol, dihydroisotanshinone I, tanshindiol, tanshin lactone, miltirone, beta-sitosterol, stigmasterol, pinosylvin, etc. |  |  |  |
|  | Angelica sinensis | Angelica sinensis (Oliv.) Diels. [Umbelliferae; Angelicae Sinensis Radix]. | Root |  |  | Grows at altitudes of 1800-2500 meters in cold and humid areas. Cultivated in Sichuan, Yunnan, Shaanxi, Guizhou, and also found in wild forests in Gansu. | Harvest in late autumn, remove fibrous roots and soil, bundle into small packages after some water evaporates, smoke-dry slowly with a fire. | Contains ligustilide, butylidenephthalide, and angelicone in volatile oil, also contains vitamin B12 and various trace elements such as iron and zinc. Additionally contains ferulic acid, phenylacetone, bisabolene, eugenol, guaiacol, anisic acid, ligustilide dimer, etc. |  |  |  |
|  | Leonurus japonicus Houtt. | Leonurus japonicus Houtt. [Lamiaceae; Leonurus japonicus Houtt.]. | Aerial parts of the plant |  |  | Grows on slopes, grasslands, and roadsides. Distributed throughout China. | Harvest when stems and leaves are flourishing, before or just after flowering, cut into sections, and sun-dry. | Whole plant contains leonurine, stachydrine, leonuridine, and leonuride diterpenoids. |  |  |  |
|  | Oldenlandia diffusa | Oldenlandia diffusa (Willd) Roxb. [Rubiaceae; Scleromitrion diffusum (Willd.) R.J.Wang]. | [Whole plant](http://www.zhongyoo.com/name/xicao_789.html) |  |  | Grows in paddy fields and moist open areas. Distributed in Guangdong, Hainan, Guangxi, etc. | Harvest in summer, wash, and use fresh or sun-dry. | Contains p-hydroxybenzoic acid, p-hydroxycinnamic acid, and linalool, 2-methyl-3-hydroxy-4-methoxyanthraquinone, scandoside methyl ester, asperuloside, etc. |  |  |  |
|  | Pyrrosia sheareri | Pyrrosia sheareri (Bak.) Ching [Polypodiaceae; Pyrrosiae Folium]. | Leaves |  |  | Usually epiphytic on rocks or tree trunks. Mainly distributed south of the Yangtze River in China. | Harvest throughout the year, remove rhizomes and fibrous roots, and air-dry or sun-dry. | Contains kaempferol, isoquercitrin, quercetin, lupeol, beta-sitosterol, mangiferin, isomangiferin, trifolin, protocatechuic acid, etc. |  |  |  |
|  | Scutellaria baicalensis Georgi | Scutellaria baicalensis Georgi [Lamiaceae; Scutellariae Radix]. | Root |  |  | Grows on sunny dry slopes, commonly found along roadsides and grassy slopes. Distributed in Yunnan, Sichuan, Guizhou, etc. | Harvest in spring and autumn, remove fibrous roots and soil, sun-dry, and remove coarse skin. | Contains flavonoids such as baicalin, baicalein, wogonoside, wogonin, oroxylin A, and minor amounts of sterols and amino acids, also contains chrysin, dihydroflavone A, etc. |  |  |  |
|  | Polyporus umbellatus | Polyporus umbellatus (Pers.) Fries [Sargassaceae; Polyporus]. | Sclerotium |  |  | Grows in cool and sunny slopes, commonly near the roots of maple, birch, oak, elm, maple, willow, and beech trees. Mainly produced in Shaanxi, Yunnan, Henan, etc. | Harvest in summer and autumn, remove soil, and sun-dry. | Contains ergosterol, biotin, proteins, crude proteins, soluble sugars, polysaccharides, and hydroxy-alpha-dihydroxy-docosanoic acid, polyporusterone A, B, C, D, E, F, G, and polyporan B. Trace elements include calcium, manganese, iron, nickel, copper, zinc, and chromium. |  |  |  |
|  | Poria cocos (Schw.) Wolf. | Poria cocos (Schw.) Wolf. [Polyporaceae; Poria]. | Sclerotium |  |  | Mostly parasitic on the roots of conifers like Pinus massoniana, P. yunnanensis, etc., at 20-30 cm underground. Distributed in Hebei, Shandong, Sichuan, etc. | Harvest throughout the year, mostly from July to September. Remove soil, pile up and cover with straw to allow water to seep out, air-dry repeatedly until dry. The core parts are sliced into pieces. | Contains triterpenoids, including poricoic acid, tumulosic acid, pachymic acid, and dehydropachymic acid. Also contains polysaccharides, mainly pachyman; histidine, adenine, choline, beta-pachymanase, protease, fatty acids, fats, lecithin, ergosterol, poria saponin, dehydropachymic acid, etc. |  |  |  |
|  | Plantago asiatica L. | Plantago asiatica L. [Plantaginaceae; Plantaginis Herba.]. | Whole plant |  |  | Grows on slopes, roadsides, field ridges, and among grass by streams. Distributed in most provinces of China. | Harvest in summer, remove soil, sun-dry or use fresh. | Contains plantain glycosides, high plantain glycosides, aucubin, ursolic acid, beta-sitosterol, stigmasterol, plantain flavonoid glycosides, eugenol glycosides, plantain glycosides, large plantain glycosides, etc. |  |  |  |
| Shen Yan Kang Fu tablets | Panax Quinquefolium | Panax quinquefolium L.[Araliaceae;Panax L.] | Dried root | Tablet | Oral, 0.3g per tablet: 8 tablets per dose, 3 times daily; 0.48g per tablet: 5 tablets per dose, 3 times daily. | Northeast China, Shaanxi Hanzhong, Shandong Weihai, Beijing Huairou. | Harvest in autumn, clean, sun-dry or dry at low temperature. | Contains saponins, polyacetylenes, flavonoids, lignans, organic acids, flavonoids, sugars, sterols, volatile oils, etc. | Tianjin Tongrentang Group Co., Ltd. | National Medicine Standard Z10940034 | Chinese Pharmacopoeia 2020 Edition Part 1 |
|  | Panax Ginseng | Panax ginseng C. A. Mey. [Araliaceae;Panax L.] | Dried root and rhizome |  |  | Liaoning, Jilin, Heilongjiang, etc. | Mostly harvested in autumn, clean, sun-dry or dry. | Contains ginsenosides, polysaccharides, amino acids, volatile oils, organic acids, etc. |  |  |  |
|  | Rehmannia Glutinosa | Rehmannia glutinosa Libosch.[Scrophulariaceae;Rehmannia Libosch. ex Fisch. et Mey.] | Fresh or dried tuberous root |  |  | Henan, Hebei, Shandong, Shanxi, etc. | Harvest in autumn, remove root heads, fibrous roots, and soil, use fresh or slowly bake until about 80% dry. | Contains iridoids and their glycosides, violetone compounds, phenylethanoid glycosides, lignans, sterols, sugars, etc. |  |  |  |
|  | Eucommia Ulmoides | Eucommia ulmoides Oliv.[Eucommiaceae;Eucommia Oliv.] | Dried bark |  |  | Shaanxi, Gansu, Henan, Hubei, Sichuan, etc. | Peel off bark from April to June, scrape off rough outer skin, stack for sweating until inner bark turns purple-brown, dry. | Contains flavonoids, phenolic acids, lignans, iridoids, steroids, etc. |  |  |  |
|  | Dioscorea Opposita | Dioscorea opposita Thunb.[Dioscoreaceae;Dioscorea L.] | Dried rhizome |  |  | Distributed in southwest China, Huaihe River Basin, and Yellow River Basin. The best variety comes from Huaiqing Prefecture in Henan, known as "Huai Yam". | Harvest after stems and leaves wither in winter, cut off root heads, wash, peel off skin and fibrous roots, dry, known as "Mao Yam"; or peel off skin, cut into thick slices while fresh, dry, known as "Yam slices"; or select plump and straight dried yams, soak in water until no dry core, roll into cylindrical shape with wooden board, sun-dry, polish, known as "Guang Yam". | Contains polysaccharides, proteins, adenosine, amino acids, trace elements, etc. |  |  |  |
|  | Scleromitrion Diffusum | Scleromitrion diffusum (Willd.) R. J. Wang[Rubiaceae;Scleromitrion] | Whole plant |  |  | Distributed in Guangdong, Hong Kong, Guangxi, Hainan, Anhui, Yunnan, etc. | Harvest in summer, wash, and use fresh or sun-dry. | Contains triterpenoids, anthraquinones, iridoids, sterols, coumarins, flavonoids, alkanes, etc. |  |  |  |
|  | Glycine Max | Glycine max（L.）Merr.[Fabaceae;Glycine] | Dried mature seeds |  |  | Cultivated in Northeast China, Loess Plateau, and other arid and semi-arid regions. | Harvest mature fruits in autumn, sun-dry, thresh, and remove impurities. | Contains flavonoids, isoflavonoids, lignans, and phenolic acids. |  |  |  |
|  | Smilax Glabra | Smilax glabra Roxb.[Smilacacea;Smilax L.] | Dried rhizome |  |  | Distributed in Guangdong, Guangxi, Anhui, Guizhou, Hunan, etc. | Harvest in summer and autumn, remove fibrous roots, wash, dry; or cut into thin slices while fresh, dry. | Contains sterols, fatty acids, phenolic glycosides, and flavonoids. |  |  |  |
|  | Leonurus Japonicus | Leonurus japonicus Houtt.[Lamiaceae;Leonurus Linn.] | Fresh or dried aerial parts |  |  | Distributed throughout China. | Harvest fresh product in spring from seedling stage to early summer before flowering; harvest dried product in summer when stems and leaves are flourishing, before or just after flowering, cut into sections, and sun-dry. | Contains alkaloids, flavonoids, diterpenes, phenylethanoid glycosides, phenolic acids, cyclic peptides, and volatile compounds. |  |  |  |
|  | Salvia Miltiorrhiza | Salvia miltiorrhiza Bge.[Lamiaceae;Salvia] | Dried root and rhizome |  |  | Shaanxi, Shandong, Shanxi, Henan, Hebei, etc. | Harvest in spring and autumn, remove soil, dry. | Contains triterpenoids, salvianolic acids. |  |  |  |
|  | Alisma Plantago-aquatica | Alisma plantago-aquatica Linn.[Alismataceae;Alisma Linn.] | Dried tuber |  |  | Sichuan, Fujian, Jiangxi, etc. | Harvest in winter when stems and leaves start to wither, wash, dry, remove fibrous roots and coarse skin. | Contains triterpenoids and sesquiterpenes. |  |  |  |
|  | Imperata Cylindrica | Imperata cylindrica Beauv.var.major（Nees）C.E.Hubb.[Poaceae Barnhart;Imperata Cyr.] | Dried rhizome |  |  | Distributed throughout China. | Harvest in spring and autumn, wash, sun-dry, remove fibrous roots and membranous leaf sheaths, bundle into small packages. | Contains triterpenoids, lactones, sterols, organic acids, etc. |  |  |  |
|  | Platycodon Grandiflorus | Platycodon grandiflorus （Jacq.）A.DC.[Campanulaceae;Platycodon A. DC.] | Dried root |  |  | Northeast China, North China, East China, etc. | Harvest in spring and autumn, wash, remove fibrous roots, peel or not peel the outer skin while fresh, dry. | Contains oleanolic acid type pentacyclic triterpenoid saponins, polysaccharides, flavonoids, phenolic compounds, sterols, fatty acids, amino acids, etc. |  |  |  |
| Qiqi Yi Shen capsules | Astragalus Membranaceus | Astragalus membranaceus（Fisch.） Bge.var.mongholicus（Bge.）Hsiao[Fabaceae;Astragalus L.] | Dried root | Capsule | Oral. 4 capsules per dose, 3 times daily. | Heilongjiang, Jilin, Liaoning, Inner Mongolia, Hebei, Shanxi, Shandong, Shaanxi, Ningxia, Gansu, Qinghai, Sichuan, Tibet, etc. | Harvest in spring and autumn, remove fibrous roots and root heads, sun-dry. | Contains saponins, polysaccharides, flavonoids, amino acids, trace elements, vitamins, etc. | Shanxi Provincial Hospital of Traditional Chinese Medicine | Shanxi Medicine Preparation No. AZ20080242 |  |
|  | Angelica Sinensis | Angelica sinensis（Oliv.）Diels[Apiaceae;Angelica L.] | Dried root |  |  | Gansu, Sichuan, Yunnan, etc. | Harvest in late autumn, remove fibrous roots and soil, bundle into small packages after some water evaporates, smoke-dry slowly with a fire. | Contains volatile oil, flavonoids, amino acids, organic acids, and polysaccharides. |  |  |  |
|  | Lycium Barbarum | Lycium barbarum L.[Solanaceae Juss.;LyciumL.] | Dried mature fruit |  |  | Ningxia, Gansu, Xinjiang, Qinghai, etc. | Harvest in summer and autumn when fruits turn red, dry with hot air, remove stems, or air-dry until skin wrinkles, sun-dry, remove stems. | Contains polysaccharides, alkaloids, flavonoids, lignans, terpenes, etc. |  |  |  |
|  | Salvia Miltiorrhiza | Salvia miltiorrhiza Bge.[Lamiaceae;Salvia] | Dried root and rhizome |  |  | Hebei, Shanxi, Shaanxi, Shandong, Henan, Jiangsu, Zhejiang, Anhui, etc. | Harvest in spring and autumn, remove soil, dry. | Contains diterpenoids, triterpenoids, phenolic acids, flavonoids, nitrogen-containing compounds, lactones, polysaccharides, etc. |  |  |  |
|  | Paeonia Lactiflora | Paeonia lactiflora Pall.[Ranunculaceae Juss.;Paeonia L.] | Dried root |  |  | Yunnan, Sichuan, Tibet, etc. | Harvest in spring and autumn, remove rhizomes, fibrous roots, and soil, sun-dry. | Contains monoterpenes and their glycosides, triterpenoids, flavonoids, tannins, phenolic acids, etc. |  |  |  |
|  | Achyranthes Bidentata | Achyranthes bidentata Bl.[Amaranthaceae;Achyranthes L.] | Dried root |  |  | Sichuan, Yunnan, Guizhou, etc. | Harvest in winter when stems and leaves wither, remove fibrous roots and soil, bundle into small packages, sun-dry until wrinkled, cut off top ends, sun-dry. | Contains saponins, sterols, polysaccharides, minor flavonoids and alkaloids. |  |  |  |
|  | Coix Lacryma-jobi | Coix lacryma-jobi L.var.ma-yuen(Roman.) Stapf[Poaceae Barnhart;Coix Linn.] | Dried mature seeds |  |  | Cultivated throughout China. | Harvest mature fruits in autumn, cut plants, sun-dry, thresh, sun-dry again, remove husks, brown seed coats, and impurities, collect seeds. | Contains fatty acids, polysaccharides, flavonoids, triterpenoids, sterols, etc. |  |  |  |
|  | Rheum Palmatum | Rheum palmatum L.[Polygonaceae;Rheum L.] | Dried root and rhizome |  |  | Qinghai, Gansu, Sichuan, Shaanxi, etc. | Harvest in late autumn or before spring sprouting when stems and leaves wither, remove fine roots, scrape off outer skin, cut into sections or pieces, string and dry, or directly dry. | Contains aloe-emodin, emodin, chrysophanol, rhein, physcion, emodin methyl ether, gallic acid, sennosides, tannins, rhubarb polysaccharides, trace elements, etc. |  |  |  |
|  | Ligusticum Chuanxiong | Ligusticum chuanxiong Hort.[Apiaceae;Ligusticum L.] | Dried rhizome |  |  | Sichuan, also in Yunnan, Guizhou, Guangxi, Hubei, etc. | Harvest in summer when stem nodes are significantly raised and slightly purple, remove soil, sun-dry and then bake dry, remove fibrous roots. | Contains phthalides, terpenes and their alcohols, alkaloids, sterols, organic acids and their esters, ginsenosides, etc. |  |  |  |
|  | Eclipta Prostrata | Eclipta prostrata L.[Asteraceae;Eclipta] | Dried aerial parts |  |  | East China, Central South, Southwest, and Liaoning, Hebei, Shaanxi, Gansu, etc. | Harvest during flowering, sun-dry. | Contains triterpenes, flavonoids, thiophenes, coumarins, lipids, sterols, etc. |  |  |  |
|  | Dioscorea Spongiosa | Dioscorea spongiosa J. Q. Xi，M. Mizuno et W. L. Zhao[Dioscoreaceae;Dioscorea L.] | Dried rhizome |  |  | Zhejiang, Jiangxi, Fujian, Hunan, Hubei, Guangdong, Guangxi, etc. | Harvest in autumn and winter, remove fibrous roots, wash, cut into slices, sun-dry. | Contains steroidal saponins, diarylheptanoids, and lignans, etc. |  |  |  |
|  | Poria Cocos | Poria cocos（Schw.）Wolf[Polyporaceae;Wolfiporia Ryv.&Gilbn] | Dried sclerotium |  |  | Hubei, Anhui, Fujian, Yunnan, Hunan, Guizhou, Sichuan, etc. | Mostly harvested from July to September, remove soil, pile up for sweating, spread out to air-dry the surface, repeat sweating and air-drying several times until wrinkles appear and most internal moisture evaporates, air-dry, known as "Poria cocos"; or cut fresh Poria cocos into different parts, air-dry, known as "Poria cocos blocks" and "Poria cocos slices". | Contains triterpenes, polysaccharides, sterols, volatile oils, proteins, amino acids, trace elements, etc. |  |  |  |
|  | Cornus Officinalis | Cornus officinalis Sieb. et Zucc.[Cornaceae;Cornus] | Dried mature fruit |  |  | Henan, Shaanxi, Zhejiang, etc. | Harvest in late autumn to early winter when the fruit skin turns red, bake with low fire or briefly blanch in boiling water, remove seeds, dry. | Contains iridoids, flavonoids, phenylpropanoids, triterpenes, etc. |  |  |  |
|  | Citrus Reticulata | Citrus reticulata Blanco [Rutaceae;Citrus L.] | Dried mature fruit peel |  |  | Guangdong, Fujian, Sichuan, Chongqing, Zhejiang, etc. | Harvest mature fruits, peel, sun-dry or low-temperature dry. | Contains flavonoids, limonin, alkaloids, organic acids, volatile oils, etc. |  |  |  |
| Huo Ba Hua Gen tablets | Tripterygium hypoglaucum (Levl.) Hutch | Tripterygium hypoglaucum (Levl.) Hutch [Celastraceae; Tripterygium wilfordii Hook. f.]. | Dried root | Tablet | Oral. 2 tablets per dose, 3 times daily. | Yunnan Chuhong, Honghe, Yuxi, Kunming, etc. | None | Contains celastrol, pigments, triterpenes, diterpenes, sesquiterpenes, alkaloids, sterols, flavonoids, sugars, tannins, etc. | Chongqing Pharmaceutical Research Institute Pharmaceutical Co., Ltd. | National Medicine Standard Z20027411 | Chinese Pharmacopoeia 2020 Edition Part One |
| Renkang Injection | Rheum Palmatum | Rheum palmatum L.[Polygonaceae;Rheum L.] | Dried root and rhizome | Injection | Intravenous infusion. 100ml per dose (5 vials, each 20ml), once daily. | Gansu, Sichuan, Qinghai, Tibet, Guizhou, Yunnan, Ningxia, etc. in high-altitude areas | Harvest in late autumn when stems and leaves wither or before spring sprouting, remove fine roots, scrape off outer skin, cut into sections or pieces, string and dry or directly dry. | Contains anthraquinones, anthrones, stilbenes, benzophenones, chromones, flavonoids, tannins, polysaccharides, volatile oils, sterols, organic acids, trace elements, etc. | Xi'an Century Shengkang Pharmaceutical Co., Ltd. | National Medicine Standard Z20040110 | State Food and Drug Administration Standard (Trial) YBZ08522004 |
|  | Astragalus Membranaceus | Astragalus membranaceus（Fisch.） Bge.var.mongholicus（Bge.）Hsiao[Fabaceae;Astragalus L.] | Dried root |  |  | Shanxi, Gansu, Inner Mongolia, Northeast China, etc. | Harvest in spring and autumn, remove fibrous roots and root heads, sun-dry. | Contains polysaccharides, saponins, flavonoids, amino acids, etc. |  |  |  |
|  | Salvia Miltiorrhiza | Salvia miltiorrhiza Bge.[Lamiaceae;Salvia] | Dried root and rhizome |  |  | Hebei, Shanxi, Shaanxi, Shandong, Henan, Jiangsu, Zhejiang, Anhui, etc. | Harvest in spring and autumn, remove soil, dry. | Contains diterpenes, triterpenes, phenolic acids, flavonoids, nitrogen-containing compounds, lactones, polysaccharides, etc. |  |  |  |
|  | Carthamus Tinctorius | Carthamus tinctorius L.[Compositae;Carthamus Linn.] | Dried flower |  |  | Xinjiang, Henan, Zhejiang, etc. | Harvest flowers in summer when they turn red, air-dry or sun-dry. | Contains chalcone quinones, flavonoids, spermidines, alkaloids, polyacetylenes, organic acids, sesquiterpenes, etc. |  |  |  |
| Huangkui capsules | Abelmoschus Manihot | Abelmoschus manihot（L.）Medic.[Malvaceae;Abelmoschus Medicus] | Dried flower crown | Capsule | Oral. 5 capsules per dose, 3 times daily. 8 weeks per course. | Distributed or cultivated in most parts of China except Northeast and Northwest regions. | Harvest flowers in summer and autumn, dry promptly. | Contains flavonoids, organic acids, volatile oils, etc. | **Suzhong Pharmaceutical Group Co., Ltd.** | National Medicine Standard Z19990040 | State Food and Drug Administration National Medicine Standard WS3-128(Z-05)-2003(Z) |

Figure S1. Sensitivity analysis of 24-hour urinary protein (HKC+BM vs BM)

Figure S2. Sensitivity analysis of 24-hour urinary protein (LGT+BM vs BM)

Figure S3. Sensitivity analysis of serum albumin (HKC+BM vs BM)

Figure S4. Sensitivity analysis of serum albumin (LGT+BM vs BM)

Figure S5. Sensitivity analysis of serum creatinine (HKC+BM vs BM)

Figure S6. Sensitivity analysis of serum creatinine (LGT+BM vs BM)

Figure S7. Sensitivity analysis of total cholesterol (LGT+BM vs BM)

Figure S8. Sensitivity analysis of triglycerides (LGT+BM vs BM)

Figure S9. Sensitivity analysis of adverse events incidence (LGT+BM vs BM)

Figure S10. Sensitivity analysis of adverse events incidence (WZC+BM vs BM)
